# Extractable Organofluorine Analysis in Pooled Human Serum and Placental Tissue Samples from an Austrian Subpopulation—A Mass Balance Analysis Approach

**DOI:** 10.1021/acs.est.1c00883

**Published:** 2021-06-16

**Authors:** Andreas-Marius Kaiser, Martin Forsthuber, Rudolf Aro, Anna Kärrman, Claudia Gundacker, Harald Zeisler, Philipp Foessleitner, Hans Salzer, Christina Hartmann, Maria Uhl, Leo W. Y. Yeung

**Affiliations:** † Environment Agency Austria, Spittelauer Lände 5, A-1090 Vienna, Austria; ‡ Institute of Medical Genetics, Center for Pathobiochemistry and Genetics, Medical University of Vienna, A-1090 Vienna, Austria; § Department of Environmental Health, Center for Public Health, Medical University of Vienna, A-1090 Vienna, Austria; ∥ Man-Technology-Environment Research Centre (MTM), Örebro University, 701 82 Örebro, Sweden; ⊥ Department of Obstetrics and Gynecology, Medical University Vienna, A-1090 Vienna, Austria; # Department of Gynecology and Obstetrics, University Hospital St. Poelten, A-3100 St. Poelten, Austria; ¶ Clinic for Pediatrics and Adolescent Medicine, University Hospital Tulln, A-3430 Tulln, Austria

**Keywords:** maternal serum, cord serum, placental tissue, per- and polyfluoroalkyl substances, unidentified organofluorine

## Abstract

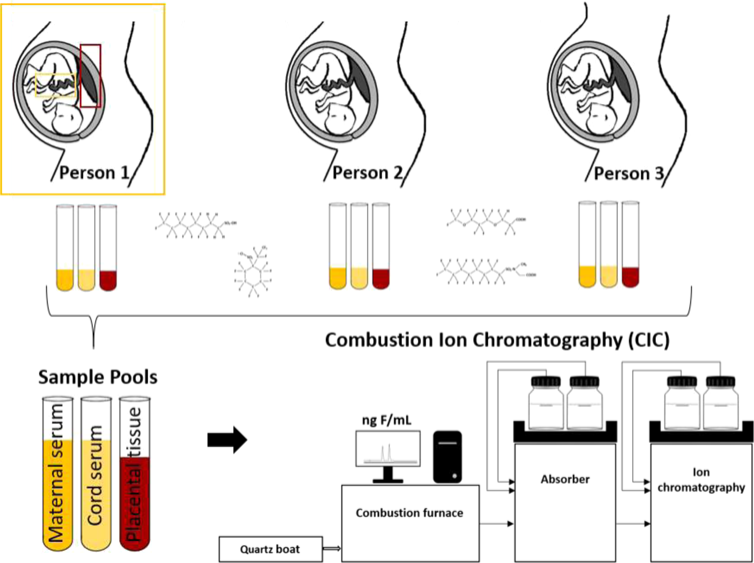

Embryos and fetuses are of major concern due to their high vulnerability. Previous studies demonstrated that human exposure to per- and polyfluoroalkyl substances (PFAS) may be underestimated because only a limited number of known PFAS can be measured. This investigation studied the total PFAS exposure by measuring the extractable organofluorine (EOF) in pooled maternal serum, placental tissue, and cord serum samples (total number of pooled samples: *n* = 45). The EOF was analyzed using combustion ion chromatography, and the concentrations of known PFAS were determined using ultraperformance liquid chromatography coupled with a tandem mass spectrometer. Using a mass balance analysis approach, the amount of unknown PFAS was estimated between the levels of known PFAS and EOF. The EOF levels ranged from 2.85 to 7.17 ng F/mL (21 PFAS were quantified) in the maternal serum, from 1.02 to 1.85 ng F/g (23 PFAS were quantified) in the placental tissue, and from 1.2 to 2.10 ng F/mL (18 PFAS were quantified) in the cord serum. An average of 24, 51, and 9% of EOF is unidentified in the maternal serum, placental tissue, and cord serum, respectively. The results show that the levels of unidentified EOF are higher in the placental tissue, suggesting accumulation or potential transformation of precursors in the placenta.

## Abstract 2

Please provide a synopsis

## 1 Introduction

Per- and polyfluoroalkyl substances (PFAS) are a group of synthetic chemicals that have been widely used for numerous industrial and commercial applications since the 1950s because of their unique chemical and physical properties, which include being hydrophobic, lipophobic, and extremely stable even at high temperatures.^1−4^ These chemicals have globally been detected in all environmental media, including biota as well as in humans.^5^ In addition to displaying persistent and bioaccumulative properties, some PFAS are suspected to cause multiple adverse health effects, for example, carcinogenicity, immunotoxicity, neurotoxicity, low birth weight in newborns, delayed puberty, and low semen quality in young men.^5−10^ The European Food Safety Authority recently set out a lower tolerable weekly intake of 4.4 ng/kg body weight based on the sum of four PFAS [perfluorooctanoic acid (PFOA), perfluorooctane sulfonate (PFOS), perfluorononanoic acid (PFNA), and perfluorohexane sulfonate (PFHxS)] due to their adverse effects on the immune system.^11^ Several studies have reported perfluoroalkyl acid (PFAA) levels in human cord serum,^12−14^ human placental tissue,^15,16^ and some even in the human embryonic and fetal tissue.^17^ Novel PFAS, which have emerged as alternatives to legacy PFAAs (e.g., PFOA and PFOS), such as per- and polyfluoroalkyl ethers [e.g., dodecalfluoro-3*H*-4,8-dioxanonanoate (ADONA)], were recently detected in human cord serum as well.^15,18,19^

In particular, exposure during the gestational period is of great concern since embryos and fetuses are generally more vulnerable to pollutants compared to adults.^20,21^ While more than 4730 PFAS-related compounds have been registered,^22^ the number of potential analytes may be far higher due to production of intermediates, degradation products, and impurities.^23^ In addition, PFAS may have multiple isomers, for example, for PFOS 89 congeners are theoretically possible.^24^ Knowledge about the toxicity of many PFAS is still limited, and additional data on human exposure might help to fill these knowledge gaps. Information on PFAS levels in the placental tissue and cord serum is a valuable contribution to a better understanding of potential developmental risks during early infancy. The study of transplacental transfer efficiencies (TTEs, comprising the ratio of PFAS levels in umbilical cord serum and in the corresponding maternal serum) is one approach to understand the toxicokinetic properties of PFAS.^13^ Previous studies of TTEs and PFAS exposure during pregnancy were limited to linear PFAS and some branched PFAAs.^13,19,25^ In these studies, concentrations of a few dozen compounds were analyzed, which is a far fewer number of PFAS to which humans might be exposed to. One method to bridge this gap is to use fluorine mass balance analysis.

Miyake et al. (2007)^26^ presented the fluorine mass balance method for estimating the overall exposure to organic fluorine compounds. This approach compares the extractable organic fluorine (EOF) levels [measured by combustion ion chromatography (CIC), for example] to the amount of fluorine from identified PFAS [quantified by target analysis, e.g., liquid chromatography coupled with tandem mass spectrometry (LC–MS/MS)] in the same sample. This method has been used to estimate the overall human exposure to PFAS.^27,28^ Studies have shown that there is a large fraction of organofluorine that is not considered by the target PFAS measured, and an increasing exposure of unidentified organofluorine was observed.^29−31^ In Germany, 0–48% of EOF levels were unidentified PFAS in plasma samples collected between 1982 and 2009, showing increasing EOF level trends since the phase-out of perfluorooctane sulfonyl fluoride-based products in the year 2000.^30^ A recent Swedish study reported a time trend for the period of 1996–2017, where only about 11–75% of EOF could be explained by target PFAS (*n* = 52); the explained EOF levels in these human serum samples declined by >3% per year.^32^ While exposures to legacy PFAS may be declining, the exposure to novel and currently unidentified PFAS was shown to be increasing.^30,32^

Knowledge on the maternal–fetal transfer of PFAS is still limited and particularly concerns EOF. Analysis of EOF in the placental tissue and cord serum can be an invaluable tool to improve the understanding of human exposure to PFAS in various biomonitoring and epidemiology studies. To our knowledge, the present study is the first to investigate EOF in the human placental tissue and cord serum.

In this study, pooled maternal serum (matS) samples (*n* = 21), cord serum (cordS) samples (*n* = 11), and placental tissue (plaT) samples (*n* = 13) from an Austrian mother-newborn study (*n* = 136) were analyzed for target PFAS and EOF. The aim of the study was (i) to determine the exposure to 61 PFAS in maternal serum, cord serum, and placental tissue and (ii) to investigate the contribution of the targeted PFAS to the EOF in all three matrices. The results of this study will contribute to a better understanding of PFAS exposure in pregnant women and their newborns and provide additional data for an improved exposure assessment.

## 2 Methods and Materials

### 2.2 Sample Collection and Sample Pooling

A total number of 136 human maternal blood, 136 placental tissue, and 136 cord blood samples were collected from 2017 to 2019 at three University Clinics (Vienna General Hospital, University Hospital Tulln, and University Hospital St. Poelten) in Eastern Austria. The samples were collected by the medical staff after written consents were obtained from the participants. The blood samples were collected with vacuum serum gel tubes (VACUETTE) and centrifuged at 1000*g* for 14 min to obtain serum. All samples were stored at −20 °C until analysis. The corresponding ethical approvals for Vienna (1035/2015) and Lower Austria (Tulln and St. Poelten) (GS1EK-4/305-2015) are available, as well as the written consent of all participants.

Individual samples were analyzed for PFAS at the Environment Agency Austria (results not shown in this publication) for another purpose. These results formed the basis for the selection of individual samples for the pooling. Due to the low concentrations detected in the samples, a selection of them showing the highest PFAS concentrations (54 maternal serum, 27 cord serum, and 32 placental tissue samples) were pooled to obtain sufficient signals for the EOF analysis.

The pooling of the maternal serum samples was based on comparable PFAS concentrations, place of residence of the mother, maternal health status, sex of the newborn, and birth outcome (i.e., small for gestational age, average for gestational age, and large for gestational age). Umbilical cord serum and placental tissue samples were pooled in the same manner. For the serum pool samples, 600 μL from two to four individuals were pooled. For placental tissue pools, individual samples were pooled using 1.5–3 g per sample. All samples from the three different matrices were pooled by considering the PFAS levels of the individual samples as well as the specific subject characteristics described above. To generate the maternal sample pools, the same participating individuals were used as for the related cord serum and placental tissue sample pools. Detailed information on the sample pools is given in Table S19 in the Supporting Information.

The final number of the pooled samples is as follows: maternal serum (*n* = 21), placental tissue (*n* = 13), and umbilical cord serum (*n* = 11). Unfortunately, volumes of the three matrices available from each individual varied, and therefore, a matched placental and cord pool for each maternal serum pool could not be achieved (see also Table S19).

### 2.1 Sample Preparation and Instrumental Analysis

The serum samples were prepared using solid phase extraction with a weak anion exchange sorbent (SPE-WAX), modified from Kuklenyik et al. (2004)^33^ and Miyake et al. (2007).^26^ The placental tissue samples were extracted with a method adapted from Martín et al. (2016)^16^ with an EnviCarb clean-up step. Details on the 61 investigated PFAS, sample preparation, and the extraction procedure are shown in the study by Kaiser et al. (2020).^34^ However, in contrast to Kaiser et al. (2020),^34^ the pooled samples were not spiked with mass labeled standards before the extraction procedure since the mass labeled standards would have contributed to the fluoride signal which would have distorted the EOF concentrations of the samples. After the extraction procedure, the sample volume was reduced to 200 μL, and the sample was split: 120 μL was used for the EOF determination; the remaining volume was then first spiked with a mass labeled standard mix, adjusted to 200 μL with the organic solvent, and subsequently prepared for the target analysis; details are given elsewhere.^34^ The target PFAS were analyzed using an ultraperformance liquid chromatography (UPLC) system from Waters (Acquity UPLC, Waters Corporation, Milford, MA, USA) coupled to either a Xevo TQ-S or a Xevo TQ-S-micro tandem mass spectrometer in the electrospray ionization negative mode. Most PFAS were analyzed using the Xevo TQ-S spectrometer, except for ADONA and hexafluoropropylene oxide dimer acid (also known as GenX), which were analyzed with the Xevo TQ-S-micro spectrometer. The analytical column was a BEH C18 1.7 μm, 2.1 × 100 mm column (Waters Corporation, Milford, MA, USA). The mobile phases used included (A) a 70:30 mixture of Milli-Q-water and methanol and (B) methanol. Both mobile phases contained 2 mmol/L ammonium acetate and 5 mmol/L *n*-methylpiperidine (the latter only for Xevo TQ-S). For PFOS, several structural isomers were analyzed. Since the baseline separation of all PFOS isomers was not achieved, they were analyzed as two clusters of branched isomers and reported as branched PFOS (br-PFOS; sum of 3-/4-/5-PFOS and 6-/2-PFOS) and linear PFOS (L-PFOS). The quantification of the PFOS isomers was based on the L-PFOS isomer standard.

The EOF content was measured with CIC (Metrohm, Switzerland) with an ion exchange column (Metrosep A Supp 5—150/4.0, Metrohm, Switzerland) and an isocratic eluent containing 64 mmol/L sodium carbonate and 20 mmol/L sodium bicarbonate in Milli-Q-water. The fluorine mass balance was performed as published in the study by Miyake et al. (2007).^26^ More details on the chemicals and instrumental analysis are provided in the study by Kaiser et al. (2020).^34^

### 2.3 Statistical Analysis and Control Measures

For the statistical analysis, R (version 3.6.3, 4.0.0, and 4.0.2) was used. The Spearman correlation coefficient (*r*_s_) was used to identify correlations between unidentified EOF and target PFAS levels. The Shapiro–Wilk test was used for testing the probability of a normal distribution, and the Levene’s test was used to assess the equality of variances. Kruskal–Wallis rank sum test was used to identify statistically significant differences in unidentified EOF rates between the three matrices. The Mann–Whitney *U* test was used to evaluate differences between the perfluoroalkyl carboxylic acid (PFCA) and perfluoroalkane sulfonic acid (PFSA) concentrations in maternal serum and cord serum samples and to further investigate statistically significant differences in PFAS concentrations between both sexes of the newborns. Differences in the TTEs between br-PFOS and L-PFOS were tested using the Wilcoxon signed-rank test. The significance level for all statistical tests was set at 0.05; to minimize the risk of false-negative findings, we reported results between 0.05 and 0.1 as marginally significant. For the statistical analysis of target PFAS, values below the limits of detection (LODs) were set to 0 and values between the LODs and the limits of quantification (LOQs) were set to LOQ/√2. Samples with EOF levels below the LOQ were excluded from the statistical analysis to avoid misinterpretations.

### 2.4 Quality Assurance and Quality Control

For the quality control assessment of the chemical analysis, accuracy was evaluated by comparing the PFAS concentrations in the pooled samples with the PFAS concentrations measured in the individual samples (data not shown). A multielement ion chromatography anion standard solution by Sigma-Aldrich (St. Louis, MO, USA) was used as the quality control material to check for inorganic fluorine contamination [see Kaiser et al. (2020)].^34^ All blanks that were spiked with inorganic fluorine to check potential bias were below the LOQ after the extraction procedure. LOD and LOQ are provided in the Supporting Information, and additional quality control procedures are described elsewhere.^34^

## 3 Results and Discussion

### 3.1 EOF Exposure in Mothers and Newborns

In this study, 21 pooled matS, 13 pooled plaT, and 11 pooled cordS samples were analyzed for 61 target PFAS and EOF. Target PFAS were used to explain the EOF and to identify the PFAS contributing to its concentration. On average, 42% of the samples investigated (29% matS, 46% plaT, and 64% cordS) showed detectable EOF levels. The LOQ varied between sample batches depending on the background of the CIC and ranged from 0.92 to 2.7 ng fluorine (F)/mL. The mean EOF concentration was 3.83 ng F/mL (ranging from 2.85 to 7.17 ng F/mL; *n* = 6) in matS, 1.26 ng F/g (ranging from 1.02 to 1.85 ng F/g; *n* = 6) in plaT, and 1.64 ng F/mL (ranging from 1.20 to 2.10 ng F/mL; *n* = 7) in cordS. To our best knowledge, this is the first study that provides data on EOF concentrations in human cord serum and placental tissue. Table 1 shows the range of EOF concentrations in maternal serum from Austria and EOF levels available from previous studies in human blood/plasma/serum.

**Table 1:** EOF Concentrations in ng F/mL in Serum from Austria and Available Previous Studies^a^

study	year of sampling	country	sample type/sample size (*n*)	S/B/P [ng F/mL]
Yeung and Mabury (2016)^30^	1982–2009	Germany^b^	plasma (42)	9.42–42.5
Miaz et al. (2020)^32^	1996–2017	Sweden^c^	serum (57)	8.1–32.0
Miyake et al., (2007b)^27^	2001	USA^b^	plasma (4)	17.8–59
Miyake et al., (2007b)^27^	2003–2004	Japan^b^	blood, serum (7)	<6–8.9
Yeung et al. (2008)^28^	2004	China^b^	blood (30)	<6–43.4
Yeung and Mabury (2016)^30^	2004	China^b^	blood (34)	8.22–94.4
**present study**	**2017–2019**	**Austria**^d^	**serum(6–7)**	**2.85–7.17**

a Earlier studies reported EOF levels in whole blood (B), plasma (P), and serum (S) samples from males and females; more details are shown in the Supporting Information.

b Extraction method: ion-pair extraction with methyl *tert*-butyl ether.

c Extraction method: acetonitrile extraction.

d Extraction method: SPE with WAX cartridges.

The ranges of EOF concentrations in the maternal serum pools are lower compared to blood/plasma/serum samples of previous studies from China,^28,30^ Germany,^30^ Sweden,^32^ the USA,^27^ and Japan.^27^ The highest EOF concentration in maternal serum in the present study was below the lowest EOF level reported from other European countries (e.g., Germany and Sweden).^30,32^ Considering the reports from Miaz et al. (2020)^32^ that the overall EOF levels remained stable in Sweden during the period from 1996 to 2017 and that no statistically significant time trends for the overall EOF levels were found in Germany,^30^ the overall EOF exposure in Austria in general is suggested to be lower compared to Sweden and Germany.

While comparing EOF results from different studies, it is very important to keep in mind that different sample preparation methods can lead to slightly different outcomes, as shown in a recent study.^34^ Moreover, apart from the applied extraction method, disparities are highly expected between matrices such as serum, plasma, and whole blood. Perfluorohexanoic acid (PFHxA), for example, is rather found in whole blood than in serum^35,36^ since the substance shows minimal binding to serum proteins.^37^ Therefore, these two factors—(1) sample preparation method and (2) sample matrix—need to be considered while comparing EOF levels between studies. Furthermore, this highlights the importance of a general standard procedure to enable unbiased EOF level comparisons between studies.

### 3.2 Unidentified EOF Exposure in Mothers and Newborns

**Figure 1:**
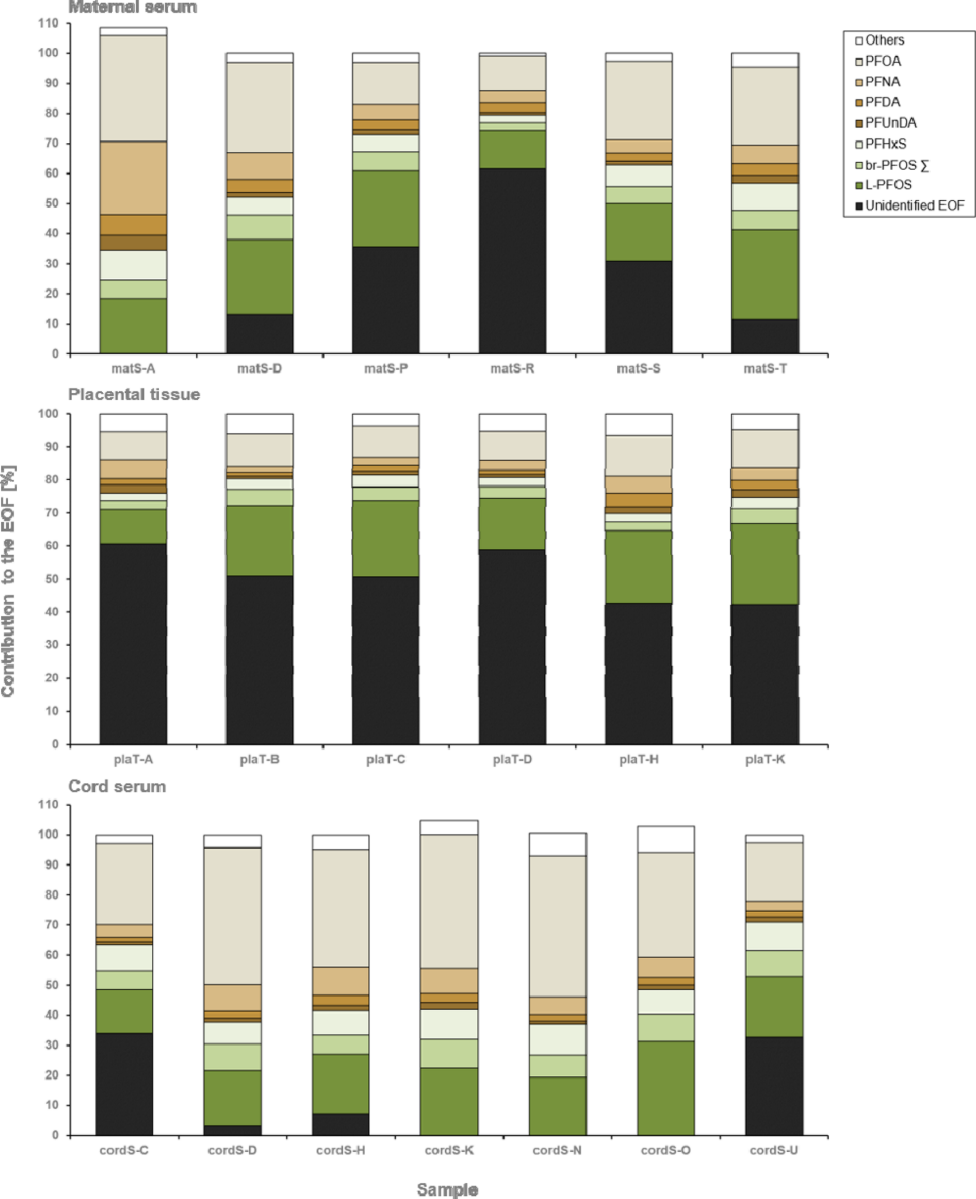
Fluorine mass balance analysis of pooled matS, pooled plaT, and pooled cordS samples with EOF levels >LOQ. Total number of samples analyzed for matS, plaT, and cordS are 21, 13, and 11, respectively.

Mass balance analysis of fluorine allows the estimation of unidentified EOF (i.e., EOF-target PFAS) in the samples. Table 2 provides a summary of the statistical parameters for identified and unidentified EOF levels for all three related matrices, and Figure 1 provides the graphical overview. There were statistically significant differences (*p* < 0.007, Kruskal–Wallis rank sum test) in unidentified EOF rates among maternal serum, placental tissue, and cord serum. Statistically, the unidentified EOF rate in cord serum was significantly lower (*p* < 0.010, posthoc: Bonferroni) compared to the placental tissue. Although this could indicate that unidentified PFAS tend to accumulate in the placenta, as mentioned in the previous section, comparisons and interpretations need to be conducted with caution when different extraction methods are applied. To avoid inter methodological biases and improve comparisons between matrices and studies, a standard operating procedure is recommended.

**Table 2:** Results of Fluorine Mass Balance Analysis for Maternal Serum, Placental Tissue, and Cord Serum Samples^a^

sample	total EOF (CIC-EOF)	identified EOF (UPLC-MS/MS-EOF)	unidentified EOF
maternal serum (*n* > LOQ = 6, total number of samples = 21)	(ng F/mL)	(ng F/mL)	(ng F/mL)
mean (±SD)	3.83 (±1.67)	2.65 (±1.40)	1.17 (±1.66)
median	3.23	2.65	0.64
min	2.85	2.08	0.0
max	7.17	3.11	4.40

sample	total EOF (CIC-EOF)	identified EOF (UPLC-MS/MS-EOF)	unidentified EOF
placental tissue (*n* > LOQ = 6, total number of samples = 13)	(ng F/g)	(ng F/g)	(ng F/g)
mean (±SD)	1.26 (±0.33)	0.60 (±0.09)	0.66 (±0.26)
median	1.08	0.58	0.55
min	1.02	0.53	0.44
max	1.85	0.77	1.08

sample	total EOF (CIC-EOF)	identified EOF (UPLC-MS/MS-EOF)	unidentified EOF
cord serum (*n* > LOQ = 7, total number of samples = 11)	(ng F/mL)	(ng F/mL)	(ng F/mL)
mean (±SD)	1.64 (±0.38)	1.49 (±0.46)	0.06 (±0.25)
median	1.80	1.24	0.0
min	1.20	1.18	0.0
max	2.10	2.23	0.60

a Sample sizes are lower compared to that in Section 3.1 since in few samples the EOF concentrations were below the LOQ of the CIC; samples with EOF levels < LOQ were not included; SD = standard deviation.

In maternal serum samples, 21 out of the 61 target PFAS monitored were present at detectable concentrations. On average, the target PFAS accounted for 76% of the EOF, and thus, approximately 24% of the EOF was of unidentified origin. The fraction of EOF remaining unidentified ranged from 0 to 61%. L-PFOS and PFOA were the biggest known drivers of the EOF exposure in the maternal serum samples, accounting for approximately 22 and 24% of the EOF, respectively. Further, br-PFOS contributed to the EOF with 5.8%. Additionally, other PFCAs [i.e., PFHxA, perfluoroheptanoic acid (PFHpA), PFNA, perfluorodecanoic acid (PFDA), perfluoroundecanoic acid (PFUnDA), and perflurorododecanoic acid (PFDoDA)] accounted for a further 15.9% of the EOF. Remaining PFSAs [i.e., perfluorobutane sulfonate, perfluoropentane sulfonate, PFHxS, and perfluoroheptane sulfonate (PFHpS)] made up another 7.9% of EOF. The remaining nine PFAS [i.e., perfluoroethylcyclohexane sulfonate (PFECHS), 3-perfluoroheptyl propanoic acid (7:3 FTCA), 2*H*-perfluoro-2-octenoic acid, 6:2 and 8:2 fluorotelomer sulfonate (FTSA), ADONA, *N*-methyl-perfluorooctane sulfonamide, *N*-methyl-perfluorooctane sulfonamido acetic acid (MeFOSAA), and *N*-ethyl-perfluorooctane sulfonamido acetic acid (EtFOSAA)] together accounted for 0.73% of the EOF in maternal serum.

Statistically significant and marginally significant positive correlations (*r*_S_ >0.75) for individual target compounds and the overall EOF concentration in maternal serum were found for 6:2 FTSA (*p* < 0.035), PFOS (*p* < 0.075), and PFECHS (*p* < 0.075). These findings could be a hint that the main source of EOF exposure in maternal serum derives from the same exposure pathways as for PFOS, PFECHS, and 6:2 FTSA. A statistically positive correlation (*r*_S_ >0.84 and *p* < 0.035) was also identified for 6:2 FTSA and the unidentified EOF concentration. A statistically negative correlation (*r*_S_ = −0.83 and *p* < 0.042) was observed for PFOA and the unidentified EOF concentration. In general, all PFCAs investigated showed negative correlations with the unidentified EOF levels. However, these correlations were not statistically significant. Comparing the individual target PFAS with the levels of total EOF and unidentified EOF suggests that unidentified PFAS are more likely to be substitutes for PFOS-related origin. A recent study by Li et al. (2020)^18^ reported the detection of 14 novel PFAS in Chinese maternal serum samples using a nontarget technique, including *p*-perfluorous noneoxybenzenesulfonate, for example, which is applied in oil production and firefighting foam.^38^ Some novel compounds reached peak areas that matched up to 105% of the peak area of PFOS in maternal serum in the semiquantitative measurements conducted.^18^

More than half of the EOF in placental tissue samples was unidentified (average: 51%), with ∑_61_PFAS accounting for 49% of the EOF on average. The fraction of EOF remaining unidentified ranged from 42 to 61%, with concentrations between 0.44 and 1.08 ng F/g. L-PFOS and PFOA made the largest contributions to the EOF levels on average, accounting for 29.5% of the EOF. br-PFOS made up an additional 3.8% of the EOF. Additionally, other PFCAs [i.e., perfluorobutanoic acid (PFBA), PFHpA, PFNA, PFDA, PFUnDA, PFDoDA, perfluorotridecanoic acid (PFTrDA), and perfluorotetradecanoic acid (PFTeDA)] accounted for further 10.7% of the EOF. The other PFSAs [i.e., PFHxS, PFHpS, and perfluorododecan sulfonate (PFDoDS)] made up another 3.3% of EOF. The remaining nine PFAS [i.e., 3-perfluoropropyl propanoic acid (3:3 FTCA), 3-perfluoropentyl propanoic acid, 7:3 FTCA, 4:2 FTSA, 8:2 FTSA, MeFOSAA, EtFOSAA, and 6:2 and 8:2 polyfluoroalkyl phosphoric acid diesters (diPAP)] accounted for 1.7% (sum) of the EOF in the placental tissue samples.

Statistically significant positive correlations (*r*_S_ >0.80) for individual targeted compounds and the overall EOF levels were found for PFBA (*p* < 0.00032), 3:3 FTCA (*p* < 0.017), MeFOSAA (*p* < 0.015), and the sum of br-PFOS (*p* < 0.037). These positive correlations are difficult to explain, but for PFBA, there might be an association with the affinity to bind on specific proteins or cells. For example, PFBA has an estimated serum half-life of 72–87 h^39^ and is usually not detected in human serum^40^ or in human urine.^40,41^ Martín et al. (2016),^16^ though, reported PFBA levels ranging between 28 and 30 ng/g in 2 out of 25 human placental tissue samples from Spain. In comparison, the present study shows PFBA levels between 0.021 and 0.053 ng/g being detected in 5 out of 13 placental tissue samples. These observations suggest that although PFBA shows a short serum half-life, it may persist in other parts of the body for a longer period than previously reported.^42^ The positive correlation between PFBA and the overall EOF may indicate that PFAS with similar physical and chemical characteristics as PFBA are more likely to accumulate in the human placenta. Statistically significant positive correlations (*r*_S_ > 0.85) were found between unidentified EOF levels and PFBA (*p* < 0.005) and MeFOSAA (*p* < 0.019). These results lead to three hypotheses: (1) unidentified PFAS may have the same exposure sources as PFBA and MeFOSAA, (2) unidentified substitutes of PFBA and MeFOSAA can be expected, and (3) MeFOSAA (which is polyfluorinated) might be biotransformed by enzymes in the placenta to unidentified PFAAs. Generally, xenobiotics can pass the placenta via passive diffusion, active transport, or to a lesser degree via metabolism.^43^ The human placenta contains multiple enzymes that can oxidize, reduce, hydrolyze, and/or conjugate foreign chemicals.^44−47^ However, the activities of those enzymes in the context of xenobiotic metabolism in the placenta are usually minor compared to those in the liver.^48^ Sulfotransferases could significantly biotransform some drugs in the placenta,^43^ which might be potential enzymes for the biotransformation of MeFOSAA. The presence of MeFOSAA, PFBA, and related compounds in the placenta might negatively influence the function of the placenta and therefore have potentially negative effects on the development of the child.^49^

In the pooled cord serum samples, on average 9% of the EOF remained unidentified (ranging from 0 to 33.4%). The majority of the identified EOF estimated based on the target PFAS analyzed (average 91%) was referred to PFCAs and PFSAs, whereas 49.4% was referred to PFCAs (36.7% specifically to PFOA) and 30.7% to PFSAs (20.8% specifically to L-PFOS). br-PFOS comprised additional 8.1% of the EOF. The other six PFAS (PFECHS, 7:3 FTCA, 6:2 FTSA, ADONA, MeFOSAA, and EtFOSAA) (sum) comprised 1.3% of the EOF in cord serum samples. While the contribution of PFCAs to the total EOF was slightly higher in the cord serum (by 9%), compared to the maternal serum, the contribution of PFSAs to the total EOF was the same in maternal and cord serum, indicating that PFCAs may pass the placenta barrier more efficiently.

No statistically significant correlations were found for any of the PFAS detected and the total EOF levels, except for PFUnDA and PFDoDA. Comparing the levels of the total EOF with the individual compounds, the Spearman correlation coefficient increased with increasing carbon-chain length of PFCAs [from C8 (*r*_S_ = 0.14) to C12 (*r*_S_ = 0.77)]. Additionally, the *p*-value decreased from C8 to C12, showing statistically significant positive correlations (*r*_S_ > 0.75) between the total EOF and PFUnDA (*p* < 0.049) and PFDoDA (p < 0.041). A possible explanation might be that longer-chain PFAS have more fluorine atoms and therefore are contributing much more to the total EOF (i.e., every additional fluoroalkyl moiety therefore increases the positive correlation coefficient). Although the same trend was not observed in maternal serum, this probably indicates that there might be a positive association between increasing fluorocarbon chains and the efficiency to pass the placenta barrier. None of the PFAS detected in the cord serum samples showed statistically significant correlations with the unidentified EOF levels, except for PFHpA which showed a statistically negative correlation (*r*_S_ = −0.85, *p* < 0.015).

### 3.3 Exposure to Individual PFAS

In total, 61 target PFAS were investigated in the present study. Out of this number, 21 were detected at least once in maternal serum samples, 23 at least once in placental tissue samples, and 18 at least once in cord serum samples (individual PFAS concentrations in detail are shown in the Supporting Information). To the best of our knowledge, this is the first time that ADONA is reported in Austrian mothers [mean: 0.022 ng/mL (±0.020)] and in their newborns [mean: 0.015 ng/mL (±0.010)]. However, ADONA has been detected in human serum (<0.2–14.4 ng/mL) in a German population at even higher concentrations close to a PFAS production site.^50^ PFECHS, in maternal serum [mean: 0.038 ng/mL (±0.0021)] as well as in cord serum [mean: 0.0027 ng/mL (±0.0019)], is also reported for the first time in an Austrian population.

An earlier study showed significant correlations of different PFAS between the paired maternal and cord serum samples.^19^ In the current investigation, positive correlations were observed, though they were not statistically significant (see Figure S1 in the Supporting Information). 6:2 diPAP and 8:2 diPAP were detected only at trace levels (below 0.040 ng/mL) in 15.4 and 7.7% of the placental tissue samples, respectively. In general, diPAPs are rarely detected in human serum or human tissue because they are quite bioactive and tend to be quickly metabolized into PFCAs upon exposure;^51^ however, Koponen et al. (2018)^52^ and Eriksson et al. (2017)^53^ detected, for example, 6:2 diPAP in serum samples from children. F53B was not detected in any of the serum or placental tissue samples, which could be explained by the lack of the historical use of this compound in Austria and the European Union. Previous studies reported the detection of F53B in maternal serum, placental tissue, and cord serum in China, which is likely due to the use of F53B in China.^15,18^

**Figure 2:**
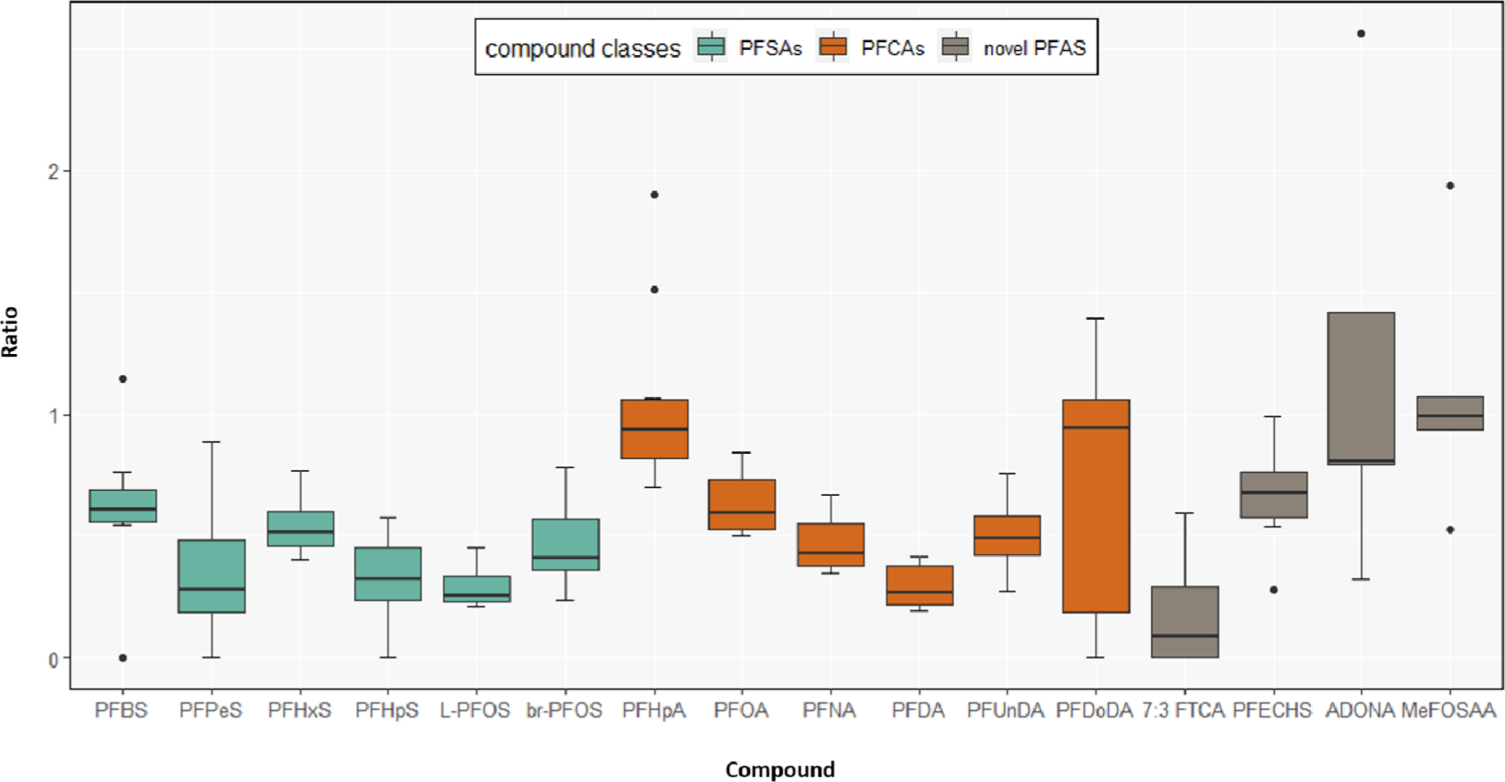
Placental transfer efficiencies (TTEs) for six perfluoroalkyl sulfonates (in green), six perfluoroalkyl carboxylates (in orange), and four novel PFAS (in gray).

Concerning the TTEs, the results of the present study support those of previous studies, which reported the transplacental transfer of some PFAAs as well as of 6:2 FTSA, 8:2 FTSA, MeFOSAA, and EtFOSAA.^14,18^ While long-chain PFAAs [e.g., PFTrDA, PFTeDA, perfluorodecanesulfonate (PFDS), and PFDoDS] and two polyfluoroalkyl phosphoric diesters (6:2 diPAP and 8:2 diPAP) were only identified in some placental tissue samples in the present study, previous studies have demonstrated that longer-chain PFAAs (e.g., PFTrDA and PFTeDA) are able to cross the placenta barrier as well, indicating that long-chain PFCAs probably do not accumulate in the placental tissue.^19^ Moreover, it is suggested that the placental transfer rate decreases with increasing perfluorinated carbon chain length until C10 according to Zhang et al. (2013)^54^ or C12 according to Li et al. (2020)^18^ and then increases with every additional perfluorinated carbon in the backbone; this results in a U-shape for placental transfer rates.^18,54^ As shown in Figure 2, the placental transfer rates in the present study decrease until PFDA and then show an increase, which confirms previous reports in the literature.^18,54^

The placental transfer for total br-PFOS isomers was higher than for L-PFOS, which is in accordance with results from earlier studies conducted by Beesoon et al. (2011)^13^ and Hanssen et al. (2013).^25^ Beesoon et al. (2011)^13^ suggested that a significant proportion of br-PFOS exposure might originate from the metabolism of PFOS precursors. Pan et al. (2017)^19^ reported that carboxylates compared to sulfonates with the same chain length were transferred through the placenta more efficiently, which is quite comparable to the present results. However, in contrast to Pan et al. (2017)^19^ and Hanssen et al. (2013),^25^ who reported that PFOS was the predominant compound in all maternal and cord serum samples, the present results show that PFOS was the predominant compound in maternal serum and PFOA was the predominant compound in cord serum. However, similar to Pan et al. (2017),^19^ no significant associations were found between PFAS concentrations and the sex of the infant. However, it has to be mentioned that the results of the present study lack in statistical power due to the small sample size (i.e., infant female = 5, infant male = 5). In contrast, statistically significant higher median concentrations of PFNA, PFDA, PFOS, and 6:2 Cl-perfluoroether sulfonic acid (PFESA) were reported in male infants compared to female infants by Wang et al. (2020).^55^ However, exposure differences between sexes in adults are, therefore, probably mostly explained by female menstruation and the disburden to the child during pregnancy and later via breastfeeding.^11,56−59^

Limitations of the study were a small sample number and low detection frequencies of EOF measurements. However, in general, the mass balance approach using the CIC is a very promising tool to address the complex issue of PFAS monitoring. As mentioned, a disadvantage that comes along using the mass balance approach is that relatively large sample volumes are still necessary to address the LOQ of CIC. This may be improved with technological progress in the future. A promising alternative that enables lower sample volumes, though, is the total oxidizable precursor (TOP) assay—a method presented by Houtz and Sedlak (2012)^60^—in which the total PFAS content is estimated by determination of PFAA concentrations before and after an oxidative process using LC–MS/MS. Further information on the total PFAS content and the suitability of both methods could be achieved by combining and comparing CIC and TOP assay results in future studies. However, the findings indicate that the EOF content cannot totally be explained via target PFAS and that a large fraction accumulates in the placenta. Furthermore, considering losses during extraction, it can be expected that the total organofluorine content is even higher than presented.

## Supporting Information Available

LOD/LOQ and concentrations of PFAS and EOF in blanks and samples and heatmap and correlation analysis (PDF)
