# Supplementary material for: Extractable Organofluorine Analysis in Pooled Human Serum and Placental Tissue Samples from an Austrian Subpopulation—A Mass Balance Analysis Approach
Source: Environ Sci Technol. 2021 Jun 16;55(13):9033–42. doi: 10.1021/acs.est.1c00883 (PMC8277134; doi:10.1021/acs.est.1c00883)
Supplement: Supplementary file 1 — es1c00883_si_001.pdf [file es1c00883_si_001.pdf]

Supplementary information (SI)

Extractable organofluorine analysis in pooled human serum and placental tissue from an Austrian sub-population – a mass balance analysis approach

Andreas-Marius Kaiser[1,2*], Martin Forsthuber[2,3], Rudolf Aro[4], Anna Kärrman[4], Claudia Gundacker[2], Harald Zeisler[5], Philipp Foessleitner[6], Hans Salzer[7], Christina Hartmann[1], Maria Uhl[1], and Leo W.Y Yeung[4**]

Affiliations

[1]Environment Agency Austria, Spittelauer Lände 5, A-1090 Vienna, Austria

[2]Institute of Medical Genetics, Center for Pathobiochemistry and Genetics, Medical University of Vienna, A-1090 Vienna, Austria

[3]Department of Environmental Health, Center for Public Health, Medical University of Vienna, A-1090 Vienna, Austria

[4]Man-Technology-Environment Research Centre (MTM), Örebro University, Örebro, 701 82, Sweden

[5] Department of Obstetrics and Gynecology, Medical University Vienna, A-1090 Vienna, Austria

[6] Department of Gynecology and Obstetrics, University Hospital St. Poelten, A-3100 St. Poelten, Austria

[7] Clinic for Pediatrics and Adolescent Medicine, University Hospital Tulln, A-3430 Tulln, Austria

Corresponding authors

*Andreas-Marius Kaiser, e-mail: Andreas.Kaiser@umweltbundesamt.at

**Leo W.Y. Yeung, e-mail: Leo.Yeung@oru.se

Table of Content

P.3 PFAS concentrations in pooled samples
P.6 Figure S1: Heatmap representing Spearman correlations […]
P.7 Limits of detection (LOD) and limits of quantification (LOQ)
P.7 Table S1: For the serum pool samples the limits of detection (LOD) […]
P.9 Table S2: For the placental tissue pool samples the limits of detection (LOD) […]
P.12 Concentrations of the individual serum and placental tissue pool samples
P.12 Table S3: PFAS concentrations of the single maternal serum pool samples matS-A to matS-E
P.14 Table S4: PFAS concentrations of the single maternal serum pool samples matS-F to matS-J
P.16 Table S5: PFAS concentrations of the single maternal serum pool samples matS-K to matS-O
P.18 Table S6: PFAS concentrations of the single maternal serum pool samples matS-P to matS-U
P.21 Table S7: PFAS concentrations of the placental pool tissue samples plaT-A to plaT-E
P.23 Table S8: PFAS concentrations of the placental pool tissue samples plaT-F to plaT-J
P.25 Table S9: PFAS concentrations of the placental pool tissue samples plaT-K to plaT-M
P.27 Table S10: PFAS concentrations of the cord serum pool samples cordS-B to cordS-H
P.30 Table S11: PFAS concentrations of the cord serum pool samples cordS-K to cordS-U
P.32 Table S12: Blanks (BW): for BW 1 and BW 2 3 mL Milli Q Water (MQ) […]
P.34 Table S13: Blanks (BW): for BW 6 and BW 7 3 mL Milli Q Water (MQ) […]
P.36 Table S14: Blanks (BW): for BW 11 and BW 12 3 mL Milli Q Water (MQ) […]
P.38 Table S15: Blanks (BW): for BW 1 and BW 3 3 mL Milli Q Water (MQ) […]
P.41 Extractable organofluorine (EOF) concentrations of the samples
P.41 Table S16: Identified EOF in ng F/mL in maternal serum (matS)
P.42 Table S17: Identified EOF in ng F/mL in placental tissue (plaT)
P.42 Table S18: Identified EOF in ng F/mL in cord serum (cordS)
P.44 Table S19: Detail information on the sample pooling
P.45 Table S20: The comparison of EOF concentrations in ng F/mL of the present study with previous studies
P.46 Correlation analyses and scatter plots
P.46 Figure S2: Correlation between PFBA and EOF-CIC, […]
P.47 Figure S3: Correlation between MeFOSAA and EOF-CIC, […]
P.48 Figure S4: Correlation between 3:3 FTCA and EOF-CIC, […]
P.49 Figure S5: Correlation between PFOS and EOF-CIC, […]
P.50 Figure S6: Correlation between 6:2 FTSA and EOF-CIC, […]
P.51 Figure S7: Correlation between PFECHS and EOF-CIC in matS
P.52 Abbreviations
P.56 References

PFAS concentrations in pooled samples

The average sum concentration (recovery corrected) of the 61 monitored PFAS ($\sum_{61}$PFAS) in maternal serum (matS) was 4.8 ng/mL (ranging from 2.7 to 9.6 ng/mL; *p* = 21), 1.6 ng/g (0.73–3.2 ng/g; *p* = 13) in placental tissue (plaT) and 2.0 ng/mL (0.35–3.3 ng/mL; *p* = 11) in cord serum (cordS).

Of the 61 PFAS included in this study, 21 were detected at least once in maternal serum samples. The PFCAs were the dominant class – making up 46.8% of the $\sum_{61}$PFAS. The PFCAs with the highest concentrations were PFOA (1.5 ng/mL (±0.89)), perfluorononanoic acid (PFNA, 0.38 ng/mL (±0.20)) and perfluorodecanoic acid (PFDA, 0.21 ng/mL (±0.13)). These compounds were found in 100% of the maternal serum samples. Perfluorohexanoic acid (PFHxA, 0.012 ng/mL (±0.0081)) and perfluoroheptanoic acid (PFHpA, 0.027 ng/mL (±0.023)) were found in 66.7% and 90.5% of the maternal serum samples. The PFSA were the second largest class of PFAS (43.2%) – driven by linear PFOS (1.6 ng/mL (±1.0)), branched PFOS (0.43 ng/mL (±0.22)), perfluorohexane sulfonate (PFHxS, 0.46 ng/mL (±0.15)) and perfluoroheptane sulfonate (PFHpS, 0.053 ng/mL (±0.030)). These compounds were found in all of the maternal serum samples. Perfluorobutane sulfonate (PFBS, 0.010 ng/mL (±0.0056)) and perfluoropentane sulfonate (PFPeS, 0.0093 ng/mL (±0.0077)) were found in 90.5% and 81.0% of the maternal serum samples. PFAA precursors together accounted for 10.0% of the $\sum_{61}$PFAS. N-methylperfluoro-n-octane sulfonamido acetic acid (MeFOSAA) and N-ethyl-perfluoro-n-octane sulfonamido acetic acid (EtFOSAA) were detected at trace levels (below 0.19 ng/mL) in 47.6% and 9.5% of samples. Of the other PFAS, perfluoro-4-ethylcyclohexane sulfonate (PFECHS) and ADONA were detected in 81.0% and 57.1% of samples respectively, with an average concentration of 0.038 ng/mL (±0.0021) and 0.022 ng/mL (±0.020).

In the placental tissue samples 23 different PFAS were detected at least once. The PFCAs were the dominant class in the placental tissue as well – making up 49.4% of the $\sum_{61}$PFAS. The PFCAs with the highest concentrations were PFOA (0.44 ng/mL (±0.31)), PFNA (0.13 ng/mL (±0.067)) and PFDA (0.068 ng/mL (±0.032)). These compounds were found in all of the placental tissue samples. Perfluorobutanoic acid (PFBA, 0.025 ng/mL (±0.018)) and PFHpA (0.011 ng/mL (±0.0064)) were found in 53.8% and 76.9% of the maternal serum samples. Long-chain PFCAs with carbon chains ranging from C11 to C14 were found in 100% of the samples except for perfluorotridecanoic acid (PFTrDA) which was detected in 92.3% of the placental tissue samples (perfluoroundecanoic acid (PFUnDA): 0.051 ng/mL (±0.030), perflurorododecanoic acid (PFDoDA): 0.024 ng/mL (±0.018), PFTrDA: 0.022 ng/mL (±0.017), perfluorotetradecanoic acid (PFTeDA): 0.039 ng/mL (±0.029)). The PFSAs were the second largest class of PFAS – linear PFOS (0.53 ng/mL (±0.26)), branched PFOS (0.094 ng/mL (±0.031)), PFHxS (0.11 ng/mL (±0.045)), PFHpS (0.014 ng/mL (±0.0067)), perfluorodecanoic sulfonate (PFDS, 0.0043 ng/mL (±0.012)) and perflurorododecanoic sulfonate (PFDoDS, 0.00060 ng/mL (±0.00020)). PFHxS, PFHpS, linear PFOS and branched PFOS were found in 100% and PFDS and PFDoDS in 7.7% of the placental tissue samples. PFAA precursors together accounted for 10.1% of the $\sum_{61}$PFAS. MeFOSAA and EtFOSAA were detected at trace levels (below 0.012 ng/mL) in 100% and 69.2% of samples. The two polyfluoroalkyl phosphoric acid diesters (diPAP), 6:2 diPAP and 8:2 diPAP, were detected at trace levels (below 0.040 ng/mL) in 15.4% and 7.7% of samples, respectively.

Of the 61 compounds monitored, 18 were found in the cord serum samples. The PFCAs were the dominant class in the cord serum samples as well – making up

56.4% of the $\sum_{61}$PFAS. The PFCAs with the highest concentrations were PFOA (0.85 ng/mL (±0.37)), PFNA (0.15 ng/mL (±0.072)) and PFDA (0.050 ng/mL (±0.023)). These compounds were found in 100% of the cord serum samples except for PFDA which was found in 90.9% of the samples. PFHxA (0.019 ng/mL (±0.0083)) and PFHpA (0.032 ng/mL (±0.022)) were found in 18.2% and 90.9% of the cord serum samples, respectively. The longer chain compounds, PFUnDA (0.034 ng/mL (±0.017)) and PFDoDA (0.010 ng/mL (±0.0060)) were found in 100% and 54.5% of the cord serum samples. The PFSAs were the second largest class of PFAS in the cord serum as well 33.3% – driven by linear PFOS (0.38 ng/mL (±0.20)), branched PFOS (0.18 ng/mL (±0.082)), PFHxS (0.22 ng/mL (±0.081)) and PFHpS (0.018 ng/mL (±0.010)). Linear and branched PFOS as well as PFHxS were found in 100% and PFHpS in 90.9% of the cord serum samples. PFBS (0.0065 ng/mL (±0.0035)) and PFPeS (0.0046 ng/mL (±0.0026)) were found both in 81.8% of cord serum samples. PFAA precursors together accounted for 10.4% of the $\sum_{61}$PFAS. MeFOSAA and EtFOSAA were detected at trace levels (below 0.17 ng/mL) in 45.5% and 18.2% of samples. Of the other PFAS, PFECHS and ADONA were detected in 90.9% and 72.7% of samples respectively, with an average concentration of 0.0027 ng/mL (±0.0019) and 0.015 ng/mL (±0.010), respectively. The limits of detection (LOD) and limits of quantification (LOQ) for the individual sample valumes are provided in Table S1 and Table S2. Table S3 to Table S11 provide invidual PFAS concentrations for all sample pools, and Table S12 to Table S15 provide the individual PFAS concentrations for all blanks.

Figure S1 shows the Spearman correlations for the pooled maternal serum and their related pooled cord serum samples (compounds at least detected in 4 related samples, except for EtFOSAA which was detected only in two related samples above

the LOD). Statistically significant positive correlations were observed for 9 PFAS, which include perfluoroheptanoic acid (PFHpA), perfluorononanoic acid (PFNA), perfluorodecanoic acid (PFDA), perfluorohexanoic sulfonate (PFHxS), L-PFOS, 7:3 FTCA, ADONA, MeFOSAA and EtFOSAA. No statistically significant differences were found in cord serum between males and females for any of the detected PFAS except for perfluoroheptanoic sulfonate (PFHpS; p <0.02).

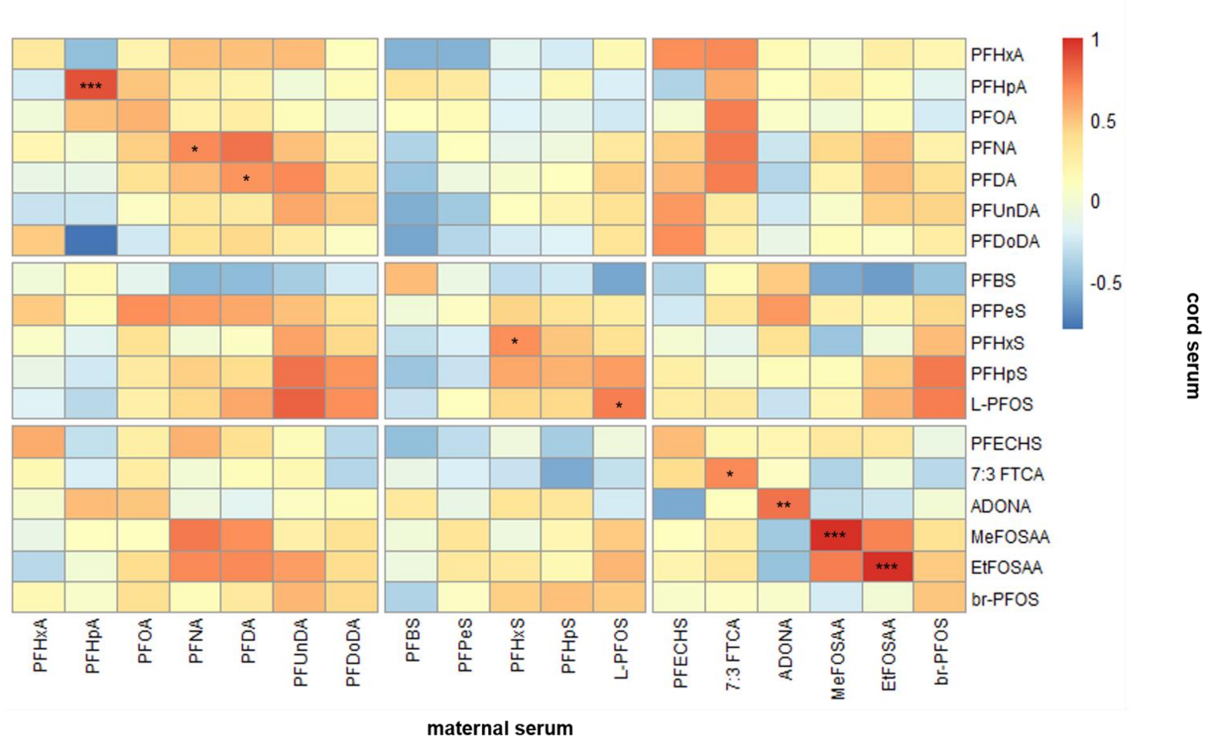

Figure S1: Heatmap representing Spearman correlations between the pooled maternal serum (*p* = 10) and their related pooled cord serum (*p* = 10), correlations that were statistically significant are marked additionally with stars (* <0.05; ** <0.01; and *** <0.001).

Limits of detection (LOD) and limits of quantification (LOQ)

Table S1: For the serum pool samples the limits of detection (LOD) and the limits of quantification (LOQ) were calculated respecting their specific sample volumes and recoveries by the applied SPE-WAX method.

**Lowest and highest LOD and lowest and highest LOQ for serum samples in pg/mL**				
**Compound**	**Lowest LOD**	**Lowest LOQ**	**Highest LOD**	**Highest LOQ**
PFBA	4.0	8.0	70	140
PFPeA	2.2	4.4	30	60
PFHxA	2.0	4.0	40	80
PFHpA	3.5	7.0	65	130
PFOA	2.5	5.0	45	90
PFNA	0.70	1.4	15	30
PFDA	6.0	12	100	200
PFUnDA	0.70	1.4	13	26
PFDoDA	2.0	4.0	35	70
PFTrDA	1.5	3.0	20	40
PFTDA	4.0	8.0	70	140
PFHxDA	65	130	950	1900
PFOcDA	48138	96277	924256	1848000
PFBS	0.30	0.60	5.0	10
PFPeS	0.30	0.60	5.0	10
PFHxS	2.5	5.0	40	80
PFHpS	0.30	0.60	5.0	10.0
L-PFOS	3.0	6.0	50	100
PFNS	0.50	1.0	8.0	16
PFDS	0.35	0.70	5.5	11
PFDoDS	7.0	14	100	200
PFECHS	0.25	0.50	4.5	9.0
FBSA	1.0	2.0	20	40
MeFBSA	8.0	16	120	240

FHxSA	0.70	1.4	11	22
MeFHxSA	2.0	4.0	35	70
FOSA	0.30	0.60	5.0	10
MeFOSA	12.5	25	250	500
EtFOSA	16	32	300	600
MeFOSE	15	30	280	560
EtFOSE	17	34	325	650
3:3 FTCA	1.6	3.2	22	44
5:3 FTCA	0.70	1.4	10	20
7:3 FTCA	0.85	1.7	12	24
6:2 FTUCA	0.80	1.6	12	24
8:2 FTUCA	0.40	0.80	6.5	13
10:2 FTUCA	0.50	1.0	8.5	17
4:2 FTSA	0.20	0.40	3.75	7.5
6:2 FTSA	0.60	1.2	7.5	15
8:2 FTSA	0.18	0.36	3.2	6.4
10:2 FTSA	5.0	10	100	200
PFHxPA	0.75	1.5	15	30
PFOPA	4800	9600	92000	184000
PFDPA	25	50	340	680
6:6 PFPiA	2.4	4.8	38	76
6:8 PFPiA	900	1800	12500	25000
8:8 PFPiA	130000	260000	2485000	4970000
8:2 Cl-PFESA	0.40	0.80	6.0	12
6:2 Cl-PFESA	0.25	0.50	3.75	7.5
ADONA	0.625	1.25	10	20
HFPO-DA (GenX)	7.5	15	100	200
MeFOSAA	1.25	2.5	25	50
EtFOSAA	5.0	10	42	84
diSAmPAP	5900	11800	47000	94000
6:2 monoPAP	43500	87000	347000	694000
8:2 monoPAP	11000	22000	87500	175000

10:2 monoPAP	14	28	100	200
6:2 diPAP	28	56	225	450
8:2 diPAP	480	960	3900	7800
6:2/8:2 diPAP	550	1100	4250	8500
10:2 diPAP	22000	44000	174000	348000
PFOS-branch ∑				

Table S2: For the placental tissue pool samples the limits of detection (LOD) and the limits of quantification (LOQ) were calculated respecting their specific sample masses and recoveries - “n.a.” means not available, since no peak was observed caused by a shift of the chromatogram.

**Lowest and highest LOD and lowest and highest LOQ for placental tissue in pg/g**				
**Compound**	**Lowest LOD**	**Lowest LOQ**	**Highest LOD**	**Highest LOQ**
PFBA	0.55	1.1	2.2	4.4
PFPeA	0.35	0.70	1.4	2.8
PFHxA	0.40	0.80	1.5	3.0
PFHpA	0.85	1.7	3.5	7.0
PFOA	0.70	1.4	3.0	6.0
PFNA	0.25	0.50	1.0	2.0
PFDA	1.8	3.6	7.0	14
PFUnDA	0.45	0.90	1.8	3.6
PFDoDA	0.40	0.80	1.5	3.0
PFTrDA	0.10	0.20	0.40	0.80
PFTDA	0.10	0.20	0.40	0.80
PFHxDA	1.4	2.8	5.5	11
PFOcDA	130	260	500	1000
PFBS	0.050	0.10	0.20	0.40
PFPeS	0.070	0.14	0.26	0.52
PFHxS	0.60	1.2	2.5	5.0
PFHpS	0.065	0.13	0.25	0.50
L-PFOS	0.50	1.0	2.0	4.0
PFNS	n.a.	n.a.	n.a.	n.a.

PFDS	0.050	0.10	0.20	0.40
PFDoDS	0.20	0.40	0.85	1.7
PFECHS	n.a.	n.a.	n.a.	n.a.
FBSA	0.20	0.40	0.80	1.6
MeFBSA	0.80	1.6	3.5	7.0
FHxSA	0.20	0.40	0.70	1.4
MeFHxSA	0.70	1.4	2.8	5.6
FOSA	0.20	0.40	0.70	1.4
MeFOSA	10	20	38	76
EtFOSA	45	90	180	360
MeFOSE	3900	7800	15500	31000
EtFOSE	n.a.	n.a.	n.a.	n.a.
3:3 FTCA	0.20	0.40	0.85	1.7
5:3 FTCA	0.075	0.15	0.30	0.60
7:3 FTCA	0.22	0.44	0.95	1.9
6:2 FTUCA	0.15	0.30	0.60	1.2
8:2 FTUCA	0.20	0.40	0.75	1.5
10:2 FTUCA	0.375	0.75	1.5	3.0
4:2 FTSA	0.030	0.060	0.12	0.24
6:2 FTSA	0.070	0.14	0.275	0.55
8:2 FTSA	0.034	0.068	0.14	0.28
10:2 FTSA	n.a.	n.a.	n.a.	n.a.
PFHxPA	0.32	0.64	1.25	2.5
PFOPA	15	30	60	120
PFDPA	30	60	120	240
6:6 PFPiA	6.0	1.2	2.5	5.0
6:8 PFPiA	100	200	430	860
8:8 PFPiA	300	600	1150	2300
8:2 Cl-PFESA	0.050	0.10	0.20	0.40
6:2 Cl-PFESA	n.a.	n.a.	n.a.	n.a.
ADONA	0.18	0.36	0.85	1.7
HFPO-DA (GenX)	90	180	350	700

MeFOSAA	0.30	0.60	1.0	2.0
EtFOSAA	1.2	2.4	5.0	10
diSAmPAP	2.0	4.0	8.25	16.5
6:2 monoPAP	7000	14000	28000	56000
8:2 monoPAP	1750	3500	7000	14000
10:2 monoPAP	1900	3800	7600	15200
6:2 diPAP	2.5	5.0	10	20
8:2 diPAP	0.75	1.5	3.0	6.0
6:2/8:2 diPAP	3.75	7.5	14	28
10:2 diPAP	250	500	1000	2000
PFOS-branch ∑				

Concentrations of the individual serum and placental tissue pool samples

Table S3: PFAS concentrations of the single maternal serum pool samples matS-A to matS-E in ng/mL (not concentrated value, so the concentrations represents the mean of each single sample in the respective pool) – the number in the “()” represents the number of samples which were pooled together

Summary of the concentrations [ng/mL] of pooled maternal serum from A-E					
**Compound**	matS-A (3)	matS-B (2)	matS-C (3)	matS-D (3)	matS-E (1)
PFBA	<LOD	<LOD	<LOD	<LOD	<LOD
PFPeA	<LOD	<LOD	<LOD	<LOD	<LOD
PFHxA	0.011	<LOQ	0.015	0.012	<LOQ
PFHpA	0.017	0.042	0.020	0.028	<LOD
PFOA	1.56	1.82	1.43	1.35	0.97
PFNA	0.98	0.23	0.34	0.36	0.56
PFDA	0.29	0.13	0.19	0.18	0.67
PFUnDA	0.19	0.060	0.063	0.045	0.38
PFDoDA	0.013	<LOD	0.011	0.0092	0.092
PFTrDA	n.a.	n.a.	n.a.	n.a.	n.a.
PFTDA	n.a.	n.a.	n.a.	n.a.	n.a.
PFHxDA	n.a.	n.a.	n.a.	n.a.	n.a.
PFOcDA	n.a.	n.a.	n.a.	n.a.	n.a.
PFBS	0.0073	0.014	0.010	0.0029	<LOD
PFPeS	0.0087	0.016	0.018	0.0054	<LOD
PFHxS	0.44	0.45	0.58	0.27	0.48
PFHpS	0.037	0.038	0.054	0.044	0.10
L-PFOS	0.77	0.93	1.49	1.07	4.63
PFNS	<LOD	<LOD	<LOD	<LOD	<LOD
PFDS	<LOD	<LOD	<LOD	<LOD	<LOD
PFDoDS	n.a.	n.a.	n.a.	n.a.	n.a.
PFECHS	<LOD	0.0032	0.0024	0.0035	<LOD
FBSA	<LOD	<LOD	<LOD	<LOD	<LOD
MeFBSA	n.a.	n.a.	n.a.	n.a.	n.a.

FHxSA	<LOD	<LOD	<LOD	<LOD	<LOD
MeFHxSA	<LOD	<LOD	<LOD	<LOD	<LOD
FOSA	<LOD	<LOD	<LOD	<LOD	<LOD
MeFOSA	<LOD	<LOD	<LOD	<LOD	<LOD
EtFOSA	<LOD	<LOD	<LOD	<LOD	<LOD
MeFOSE	<LOD	<LOD	<LOD	<LOD	<LOD
EtFOSE	<LOD	<LOD	<LOD	<LOD	<LOD
3:3 FTCA	<LOD	<LOD	<LOD	<LOD	<LOD
5:3 FTCA	<LOD	<LOD	<LOD	<LOD	<LOD
7:3 FTCA	0.016	0.012	<LOD	0.0068	<LOD
6:2 FTUCA	<LOD	<LOD	<LOD	<LOD	<LOD
8:2 FTUCA	<LOD	<LOD	<LOD	<LOD	<LOD
10:2 FTUCA	<LOD	<LOD	<LOD	<LOD	<LOD
4:2 FTSA	<LOD	<LOD	<LOD	<LOD	<LOD
6:2 FTSA	n.a.	n.a.	n.a.	n.a.	n.a.
8:2 FTSA	<LOD	<LOD	<LOD	<LOD	<LOD
10:2 FTS	<LOD	<LOD	<LOD	<LOD	<LOD
PFHxPA	<LOD	<LOD	<LOD	<LOD	<LOD
PFOPA	n.a.	n.a.	n.a.	n.a.	n.a.
PFDPA	n.a.	n.a.	n.a.	n.a.	n.a.
6:6 PFPiA	<LOD	<LOD	<LOD	<LOD	<LOD
6:8 PFPiA	n.a.	n.a.	n.a.	n.a.	n.a.
8:8 PFPiA	n.a.	n.a.	n.a.	n.a.	n.a.
8:2 Cl-PFESA	<LOD	<LOD	<LOD	<LOD	<LOD
6:2 Cl-PFESA	<LOD	<LOD	<LOD	<LOD	<LOD
ADONA	<LOD	0.0056	0.024	<LOD	0.0736
HFPO-DA (GenX)	n.a.	n.a.	n.a.	n.a.	n.a.
MeFOSAA	0.0058	<LOD	<LOQ	0.012	<LOD
EtFOSAA	<LOD	<LOD	<LOD	<LOD	<LOD
diSAmPAP	n.a.	n.a.	n.a.	n.a.	n.a.
6:2 monoPAP	n.a.	n.a.	n.a.	n.a.	n.a.
8:2 monoPAP	n.a.	n.a.	n.a.	n.a.	n.a.

10:2 monoPAP	<LOD	<LOD	<LOD	<LOD	<LOD
6:2 diPAP	<LOD	<LOD	<LOD	<LOD	<LOD
8:2 diPAP	n.a.	n.a.	n.a.	n.a.	n.a.
6:2/8:2 diPAP	n.a.	n.a.	n.a.	n.a.	n.a.
10:2 diPAP	n.a.	n.a.	n.a.	n.a.	n.a.
PFOS-branch ∑	0.28	0.30	0.50	0.36	0.50

Table S4: PFAS concentrations of the single maternal serum pool samples matS-F to matS-J in ng/mL (not concentrated value, so the concentrations represents the mean of each single sample in the respective pool) – the number in the “()” represents the number of samples which were pooled together

**Summary of the concentrations [ng/mL] of pooled maternal serum from F-M**					
**Compound**	matS-F (4)	matS-G (2)	matS-H (3)	matS-I (1)	matS-J (2)
PFBA	<LOD	<LOD	<LOD	<LOD	<LOD
PFPeA	<LOD	<LOD	<LOD	<LOD	<LOD
PFHxA	0.011	0.025	<LOQ	<LOD	0.016
PFHpA	0.012	<LOQ	<LOQ	<LOD	0.056
PFOA	0.78	0.52	1.41	3.62	4.17
PFNA	0.21	0.15	0.50	0.64	0.51
PFDA	0.12	0.048	0.30	0.23	0.34
PFUnDA	0.043	0.010	0.080	0.033	0.14
PFDoDA	0.0071	0.0040	0.011	<LOD	0.042
PFTrDA	n.a.	n.a.	n.a.	n.a.	n.a.
PFTDA	n.a.	n.a.	n.a.	n.a.	n.a.
PFHxDA	n.a.	n.a.	n.a.	n.a.	n.a.
PFOcDA	n.a.	n.a.	n.a.	n.a.	n.a.
PFBS	0.0063	0.011	0.0073	0.010	0.022
PFPeS	0.0040	0.011	0.0038	<LOD	0.0051
PFHxS	0.25	0.72	0.39	0.59	0.74
PFHpS	0.034	0.068	0.033	0.075	0.061
L-PFOS	0.91	1.27	1.45	1.05	2.72
PFNS	<LOD	<LOD	<LOD	<LOD	<LOD

PFDS	<LOD	<LOD	<LOD	<LOD	<LOD
PFDoDS	n.a.	n.a.	n.a.	n.a.	n.a.
PFECHS	0.0023	n.n.	0.011	n.n.	0.0055
FBSA	<LOD	<LOD	<LOD	<LOD	<LOD
MeFBSA	n.a.	n.a.	n.a.	n.a.	n.a.
FHxSA	<LOD	<LOD	<LOD	<LOD	<LOD
MeFHxSA	<LOD	<LOD	<LOD	<LOD	<LOD
FOSA	<LOD	<LOD	<LOD	<LOD	<LOD
MeFOSA	<LOD	<LOD	<LOD	<LOD	<LOD
EtFOSA	<LOD	<LOD	<LOD	<LOD	<LOD
MeFOSE	<LOD	<LOD	<LOD	<LOD	<LOD
EtFOSE	<LOD	<LOD	<LOD	<LOD	<LOD
3:3 FTCA	<LOD	<LOD	<LOD	<LOD	<LOD
5:3 FTCA	<LOD	<LOD	<LOD	<LOD	<LOD
7:3 FTCA	0.0040	<LOD	0.013	<LOD	<LOD
6:2 FTUCA	<LOD	<LOD	<LOD	<LOD	<LOD
8:2 FTUCA	<LOD	<LOD	<LOD	<LOD	<LOD
10:2 FTUCA	<LOD	<LOD	<LOD	<LOD	<LOD
4:2 FTSA	<LOD	<LOD	<LOD	<LOD	<LOD
6:2 FTSA	n.a.	n.a.	n.a.	n.a.	n.a.
8:2 FTSA	<LOD	<LOD	<LOD	<LOD	<LOD
10:2 FTSA	<LOD	<LOD	<LOD	<LOD	<LOD
PFHxPA	<LOD	<LOD	<LOD	<LOD	<LOD
PFOPA	n.a.	n.a.	n.a.	n.a.	n.a.
PFDPA	n.a.	n.a.	n.a.	n.a.	n.a.
6:6 PFPiA	<LOD	<LOD	<LOD	<LOD	<LOD
6:8 PFPiA	n.a.	n.a.	n.a.	n.a.	n.a.
8:8 PFPiA	n.a.	n.a.	n.a.	n.a.	n.a.
8:2 Cl-PFESA	<LOD	<LOD	<LOD	<LOD	<LOD
6:2 Cl-PFESA	<LOD	<LOD	<LOD	<LOD	<LOD
ADONA	0.0071	0.0060	<LOD	<LOD	<LOQ
HFPO-DA (GenX)	n.a.	n.a.	n.a.	n.a.	n.a.

MeFOSAA	0.028	<LOD	0.020	<LOD	<LOD
EtFOSAA	<LOD	<LOD	<LOQ	<LOD	<LOD
diSAmPAP	n.a.	n.a.	n.a.	n.a.	n.a.
6:2 monoPAP	n.a.	n.a.	n.a.	n.a.	n.a.
8:2 monoPAP	n.a.	n.a.	n.a.	n.a.	n.a.
10:2 monoPAP	<LOD	<LOD	<LOD	<LOD	<LOD
6:2 diPAP	<LOD	<LOD	<LOD	<LOD	<LOD
8:2 diPAP	n.a.	n.a.	n.a.	n.a.	n.a.
6:2/8:2 diPAP	n.a.	n.a.	n.a.	n.a.	n.a.
10:2 diPAP	n.a.	n.a.	n.a.	n.a.	n.a.
PFOS-branch ∑	0.23	0.36	0.38	0.60	0.59

Table S5: PFAS concentrations of the single maternal serum pool samples matS-K to matS-O in ng/mL (not concentrated value, so the concentrations represents the mean of each single sample in the respective pool) – the number in the “()” represents the number of samples which were pooled together

**Summary of the concentrations [ng/mL] of pooled maternal serum from K-O**					
**Compound**	matS-K (3)	matS-L (3)	matS-M (2)	matS-N (3)	matS-O (2)
PFBA	<LOD	<LOD	<LOD	<LOD	<LOD
PFPeA	<LOD	<LOD	<LOD	<LOD	<LOD
PFHxA	0.012	0.0088	<LOD	<LOD	<LOD
PFHpA	0.021	0.026	<LOQ	0.090	0.075
PFOA	1.69	1.50	1.47	1.38	2.11
PFNA	0.44	0.23	0.27	0.27	0.65
PFDA	0.22	0.12	0.15	0.12	0.31
PFUnDA	0.096	0.050	0.052	0.049	0.11
PFDoDA	0.013	<LOD	<LOD	0.014	0.050
PFTrDA	n.a.	n.a.	n.a.	n.a.	n.a.
PFTDA	n.a.	n.a.	n.a.	n.a.	n.a.
PFHxDA	n.a.	n.a.	n.a.	n.a.	n.a.

PFOcDA	n.a.	n.a.	n.a.	n.a.	n.a.
PFBS	<LOD	0.0078	0.0062	0.018	0.0096
PFPeS	<LOD	<LOQ	<LOD	0.010	0.032
PFHxS	0.44	0.54	0.48	0.33	0.68
PFHpS	0.046	0.038	0.032	0.058	0.16
L-PFOS	1.38	0.86	1.51	1.02	3.97
PFNS	<LOD	<LOD	<LOD	<LOD	<LOD
PFDS	<LOD	<LOD	<LOD	<LOD	<LOD
PFDoDS	n.a.	n.a.	n.a.	n.a.	n.a.
PFECHS	0.0051	0.0033	<LOQ	0.0022	0.0033
FBSA	<LOD	<LOD	<LOD	<LOD	<LOD
MeFBSA	n.a.	n.a.	<LOD	<LOD	<LOD
FHxSA	<LOD	<LOD	<LOD	<LOD	<LOD
MeFHxSA	<LOD	<LOD	<LOD	<LOD	<LOD
FOSA	<LOD	<LOD	<LOD	<LOD	<LOD
MeFOSA	<LOD	<LOD	<LOD	<LOD	<LOD
EtFOSA	<LOD	<LOD	n.a.	n.a.	n.a.
MeFOSE	<LOD	<LOD	<LOD	<LOD	<LOD
EtFOSE	<LOD	<LOD	<LOD	<LOD	<LOD
3:3 FTCA	<LOD	<LOD	<LOD	<LOD	<LOD
5:3 FTCA	<LOD	<LOD	<LOD	<LOD	<LOD
7:3 FTCA	0.021	<LOD	0.0086	0.010	0.0097
6:2 FTUCA	<LOD	<LOD	<LOQ	<LOD	<LOD
8:2 FTUCA	<LOD	<LOD	<LOD	<LOD	<LOD
10:2 FTUCA	<LOD	<LOD	<LOD	<LOD	<LOD
4:2 FTSA	<LOD	<LOD	<LOD	<LOD	<LOD
6:2 FTSA	n.a.	n.a.	<LOD	<LOD	<LOD
8:2 FTSA	<LOD	<LOD	<LOD	0.0060	<LOD
10:2 FTSA	<LOD	<LOD	n.a.	n.a.	n.a.
PFHxPA	<LOD	<LOD	<LOD	<LOD	<LOD
PFOPA	n.a.	n.a.	n.a.	n.a.	n.a.
PFDPA	n.a.	n.a.	n.a.	n.a.	n.a.

6:6 PFPiA	<LOD	<LOD	<LOD	<LOD	<LOD
6:8 PFPiA	n.a.	n.a.	n.a.	n.a.	n.a.
8:8 PFPiA	n.a.	n.a.	n.a.	n.a.	n.a.
8:2 Cl-PFESA	<LOD	<LOD	<LOD	<LOD	<LOD
6:2 Cl-PFESA	<LOD	<LOD	<LOD	<LOD	<LOD
ADONA	0.027	0.033	0.041	0.019	<LOD
HFPO-DA (GenX)	n.a.	n.a.	n.a.	n.a.	n.a.
N-MeFOSAA	<LOD	<LOD	<LOQ	<LOQ	0.030
N-EtFOSAA	<LOD	<LOD	<LOD	<LOD	0.19
diSAmPAP	n.a.	n.a.	n.a.	n.a.	n.a.
6:2 monoPAP	n.a.	n.a.	n.a.	n.a.	n.a.
8:2 monoPAP	n.a.	n.a.	n.a.	n.a.	n.a.
10:2 monoPAP	<LOD	<LOD	<LOD	<LOD	<LOD
6:2 diPAP	<LOD	<LOD	<LOD	<LOD	<LOD
8:2 diPAP	n.a.	n.a.	n.a.	n.a.	n.a.
6:2/8:2 diPAP	n.a.	n.a.	n.a.	n.a.	n.a.
10:2 diPAP	n.a.	n.a.	n.a.	n.a.	n.a.
PFOS-branch ∑	0.40	0.33	0.52	0.37	1.22

Table S6: PFAS concentrations of the single maternal serum pool samples matS-P to matS-U in ng/mL (not concentrated value, so the concentrations represents the mean of each single sample in the respective pool) – the number in the “()” represents the number of samples which were pooled together

**Summary of the concentrations [ng/mL] of pooled maternal serum from P-U**						
**Compound**	matS-P (3)	matS-Q (3)	matS-R (3)	matS-S (3)	matS-T (3)	matS-U (2)
PFBA	<LOD	<LOD	<LOD	<LOD	<LOD	<LOD
PFPeA	<LOD	<LOD	<LOD	<LOD	<LOD	<LOD
PFHxA	<LOQ	<LOQ	<LOD	<LOD	<LOQ	<LOD
PFHpA	0.013	0.013	<LOQ	0.021	0.031	<LOQ
PFOA	0.72	0.73	1.20	1.12	1.29	0.94
PFNA	0.27	0.24	0.41	0.19	0.31	0.22
PFDA	0.15	0.14	0.30	0.096	0.18	0.12

PFUnDA	0.072	0.081	0.078	0.042	0.12	0.078
PFDoDA	0.017	<LOD	<LOD	<LOD	0.017	0.023
PFTrDA	n.a.	n.a.	n.a.	n.a.	n.a.	n.a.
PFTDA	n.a.	n.a.	n.a.	n.a.	n.a.	n.a.
PFHxDA	n.a.	n.a.	n.a.	n.a.	n.a.	n.a.
PFOcDA	n.a.	n.a.	n.a.	n.a.	n.a.	n.a.
PFBS	0.0074	0.0090	0.0042	0.017	0.021	0.0040
PFPeS	0.0053	0.0034	0.0044	0.012	0.016	0.0037
PFHxS	0.30	0.38	0.26	0.32	0.45	0.49
PFHpS	0.043	0.031	0.020	0.033	0.042	0.061
L-PFOS	1.20	0.96	1.21	0.76	1.38	2.07
PFNS	<LOD	<LOD	<LOD	<LOD	<LOD	<LOD
PFDS	<LOD	<LOD	<LOD	<LOD	<LOD	<LOD
PFDoDS	n.a.	n.a.	n.a.	n.a.	n.a.	n.a.
PFECHS	0.0043	0.0029	0.0034	0.0034	0.0050	0.0034
FBSA	<LOD	<LOD	<LOD	<LOD	<LOD	<LOD
MeFBSA	<LOD	<LOD	<LOD	<LOD	<LOD	<LOD
FHxSA	<LOD	<LOD	<LOD	<LOD	<LOD	<LOD
MeFHxSA	<LOD	<LOD	<LOD	<LOD	<LOD	<LOD
FOSA	<LOD	<LOD	<LOD	<LOD	<LOD	<LOD
MeFOSA	<LOD	<LOD	<LOD	<LOD	<LOD	<LOD
EtFOSA	n.a.	n.a.	n.a.	n.a.	n.a.	n.a.
MeFOSE	<LOD	<LOD	<LOD	<LOD	0.14	<LOD
EtFOSE	<LOD	<LOD	<LOD	<LOD	<LOD	<LOD
3:3 FTCA	<LOD	<LOD	<LOD	<LOD	<LOD	<LOD
5:3 FTCA	<LOD	<LOD	<LOD	<LOD	<LOD	<LOD
7:3 FTCA	<LOQ	<LOQ	0.010	<LOQ	0.0051	<LOD
6:2 FTUCA	<LOD	<LOD	0.0054	<LOD	<LOQ	<LOD
8:2 FTUCA	<LOD	<LOD	<LOD	<LOD	<LOD	<LOD
10:2 FTUCA	<LOD	<LOD	<LOD	<LOD	<LOD	<LOD
4:2 FTSA	<LOD	<LOD	<LOD	<LOD	<LOD	<LOD
6:2 FTSA	0.0020	<LOD	0.0057	<LOD	<LOD	<LOD

8:2 FTSA	0.016	<LOD	<LOD	<LOD	<LOD	<LOD
10:2 FTSA	n.a.	n.a.	n.a.	n.a.	n.a.	n.a.
PFHxPA	<LOD	<LOD	<LOD	<LOD	<LOD	<LOD
PFOPA	n.a.	n.a.	n.a.	n.a.	n.a.	n.a.
PFDPA	n.a.	n.a.	n.a.	n.a.	n.a.	n.a.
6:6 PFPiA	<LOD	<LOD	<LOD	<LOD	<LOD	<LOD
6:8 PFPiA	n.a.	n.a.	n.a.	n.a.	n.a.	n.a.
8:8 PFPiA	n.a.	n.a.	n.a.	n.a.	n.a.	n.a.
8:2 Cl-PFESA	<LOD	<LOD	<LOD	<LOD	<LOD	<LOD
6:2 Cl-PFESA	<LOD	<LOD	<LOD	<LOD	<LOD	<LOD
ADONA	<LOD	<LOD	0.0072	<LOD	0.020	<LOD
HFPO-DA (GenX)	n.a.	n.a.	n.a.	n.a.	n.a.	n.a.
MeFOSAA	0.011	0.017	<LOD	<LOD	<LOD	<LOD
EtFOSAA	<LOD	<LOD	<LOD	<LOD	<LOD	<LOD
diSAmPAP	n.a.	n.a.	n.a.	n.a.	n.a.	n.a.
6:2 monoPAP	n.a.	n.a.	n.a.	n.a.	n.a.	n.a.
8:2 monoPAP	n.a.	n.a.	n.a.	n.a.	n.a.	n.a.
10:2 monoPAP	<LOD	<LOD	<LOD	<LOD	<LOD	<LOD
6:2 diPAP	<LOD	<LOD	<LOD	<LOD	<LOD	<LOD
8:2 diPAP	n.a.	n.a.	n.a.	n.a.	n.a.	n.a.
6:2/8:2 diPAP	n.a.	n.a.	n.a.	n.a.	n.a.	n.a.
10:2 diPAP	n.a.	n.a.	n.a.	n.a.	n.a.	n.a.
PFOS-branch ∑	0.35	0.21	0.28	0.25	0.34	0.68

Table S7: PFAS concentrations of the placental pool tissue samples plaT-A to plaT-E in ng/g (not concentrated value, so the concentrations represents the mean of each single sample in the respective pool) – the number in the "()" represents the number of samples which were pooled together

**Summary of the concentrations [ng/g] of pooled placental tissue from A-E**					
**Compound**	plaT-A (3)	plaT-B (2)	plaT-C (3)	plaT-D (3)	plaT-E (1)
PFBA	0.031	0.029	<LOD	0.053	<LOD
PFPeA	<LOD	<LOD	<LOD	<LOD	<LOD
PFHxA	<LOD	<LOD	<LOD	<LOD	<LOD
PFHpA	<LOD	0.0095	0.0077	0.013	0.0038
PFOA	0.35	0.31	0.29	0.47	0.14
PFNA	0.26	0.056	0.085	0.17	0.11
PFDA	0.077	0.031	0.047	0.069	0.12
PFUnDA	0.11	0.024	0.035	0.043	0.10
PFDoDA	0.027	0.013	0.013	0.022	0.070
PFTrDA	0.032	0.016	0.018	0.0049	0.032
PFTDA	0.029	0.023	0.020	0.028	0.090
PFHxDA	n.a.	n.a.	n.a.	n.a.	n.a.
PFOcDA	n.a.	n.a.	n.a.	n.a.	n.a.
PFBS	<LOD	<LOD	<LOD	<LOD	<LOD
PFPeS	<LOD	<LOD	<LOD	<LOD	<LOD
PFHxS	0.080	0.080	0.098	0.13	0.060
PFHpS	0.0076	0.013	0.012	0.016	0.012
L-PFOS	0.27	0.41	0.44	0.52	0.98
PFNS	<LOD	<LOD	n.a.	n.a.	n.a.
PFDS	<LOD	<LOD	<LOD	<LOD	0.0043
PFDoDS	0.0006	<LOD	<LOD	<LOD	<LOD
PFECHS	n.a.	n.a.	n.a.	n.a.	n.a.
FBSA	<LOD	<LOD	<LOD	<LOD	<LOD
MeFBSA	<LOD	<LOD	<LOD	<LOD	<LOD
FHxSA	<LOD	<LOD	<LOD	<LOD	<LOD
MeFHxSA	<LOD	<LOD	<LOD	<LOD	<LOD

FOSA	n.a.	n.a.	n.a.	n.a.	n.a.
MeFOSA	n.a.	n.a.	n.a.	n.a.	n.a.
EtFOSA	n.a.	n.a.	n.a.	n.a.	n.a.
MeFOSE	n.a.	n.a.	n.a.	n.a.	n.a.
EtFOSE	n.a.	n.a.	n.a.	n.a.	n.a.
3:3 FTCA	<LOD	0.0007	0.0006	0.0014	<LOD
5:3 FTCA	<LOD	<LOD	<LOD	0.0013	<LOD
7:3 FTCA	0.012	0.012	0.0027	0.035	0.011
6:2 FTUCA	<LOD	<LOD	<LOD	<LOD	<LOD
8:2 FTUCA	n.a.	n.a.	n.a.	n.a.	n.a.
10:2 FTUCA	n.a.	n.a.	n.a.	n.a.	n.a.
4:2 FTSA	0.0098	0.0054	0.0065	0.0088	0.0028
6:2 FTSA	<LOD	<LOD	<LOD	<LOD	<LOD
8:2 FTSA	0.013	0.0068	<LOD	<LOD	0.0042
10:2 FTSA	n.a.	n.a.	n.a.	n.a.	n.a.
PFHxPA	<LOD	<LOD	<LOD	<LOD	<LOD
PFOPA	n.a.	n.a.	n.a.	n.a.	n.a.
PFDPA	n.a.	n.a.	n.a.	n.a.	n.a.
6:6 PFPiA	<LOD	<LOD	<LOD	<LOD	<LOD
6:8 PFPiA	n.a.	n.a.	n.a.	n.a.	n.a.
8:8 PFPiA	n.a.	n.a.	n.a.	n.a.	n.a.
8:2 Cl-PFESA	<LOD	<LOD	<LOD	<LOD	<LOD
6:2 Cl-PFESA	n.a.	n.a.	n.a.	n.a.	n.a.
ADONA	<LOD	<LOD	<LOD	<LOD	<LOD
HFPO-DA (GenX)	n.a.	n.a.	n.a.	n.a.	n.a.
MeFOSAA	0.0076	0.0088	0.0051	0.017	0.0044
EtFOSAA	<LOD	0.0062	0.0036	0.0066	0.0033
diSAmPAP	<LOD	<LOD	<LOD	<LOD	<LOD
6:2 monoPAP	n.a.	n.a.	n.a.	n.a.	n.a.
8:2 monoPAP	n.a.	n.a.	n.a.	n.a.	n.a.
10:2 monoPAP	n.a.	n.a.	n.a.	n.a.	n.a.
6:2 diPAP	<LOD	<LOD	<LOD	0.0049	<LOD

8:2 diPAP	<LOD	<LOD	<LOD	0.0022	<LOD
6:2/8:2 diPAP	<LOD	<LOD	<LOD	<LOD	<LOD
10:2 diPAP	n.a.	n.a.	n.a.	n.a.	n.a.
PFOS-branch ∑	0.066	0.096	0.081	0.12	0.10

Table S8: PFAS concentrations of the placental pool tissue samples plaT-F to plaT-J in ng/g (not concentrated value, so the concentrations represents the mean of each single sample in the respective pool) – the number in the “()” represents the number of samples which were pooled together

**Summary of the concentrations [ng/g] of pooled placental tissue from F-J**					
**Compound**	plaT-F (4)	plaT-G (2)	plaT-H (3)	plaT-I (1)	plaT-J (2)
PFBA	0.021	<LOD	<LOD	<LOD	<LOD
PFPeA	<LOD	<LOD	<LOD	<LOD	<LOD
PFHxA	<LOD	<LOD	<LOD	<LOD	<LOD
PFHpA	0.0048	<LOD	0.0090	0.010	0.019
PFOA	0.14	0.21	0.36	1.00	1.15
PFNA	0.058	0.073	0.16	0.25	0.16
PFDA	0.026	0.041	0.12	0.087	0.095
PFUnDA	0.020	0.030	0.061	0.035	0.073
PFDoDA	0.0099	0.013	0.026	0.013	0.050
PFTrDA	0.0099	0.0091	0.019	0.0035	0.064
PFTDA	0.021	0.021	0.039	0.017	0.11
PFHxDA	n.a.	n.a.	n.a.	n.a.	n.a.
PFOcDA	n.a.	n.a.	n.a.	n.a.	n.a.
PFBS	<LOD	<LOD	<LOD	<LOD	<LOD
PFPeS	<LOD	<LOD	<LOD	<LOD	<LOD
PFHxS	0.037	0.17	0.059	0.15	0.18
PFHpS	0.0058	0.026	0.0075	0.020	0.025
L-PFOS	0.28	0.86	0.39	0.49	1.07
PFNS	n.a.	n.a.	n.a.	n.a.	n.a.

PFDS	<LOD	<LOD	<LOD	<LOD	<LOD
PFDoDS	<LOD	<LOD	<LOD	<LOD	<LOD
PFECHS	n.a.	n.a.	n.a.	n.a.	n.a.
FBSA	<LOD	<LOD	<LOD	<LOD	<LOD
MeFBSA	<LOD	<LOD	<LOD	<LOD	<LOD
FHxSA	<LOD	<LOD	<LOD	<LOD	<LOD
MeFHxSA	<LOD	<LOD	<LOD	<LOD	<LOD
FOSA	n.a.	n.a.	n.a.	n.a.	n.a.
MeFOSA	n.a.	n.a.	n.a.	n.a.	n.a.
EtFOSA	n.a.	n.a.	n.a.	n.a.	n.a.
MeFOSE	n.a.	n.a.	n.a.	n.a.	n.a.
EtFOSE	n.a.	n.a.	n.a.	n.a.	n.a.
3:3 FTCA	0.0005	<LOD	<LOD	<LOD	<LOD
5:3 FTCA	<LOD	<LOD	<LOD	<LOD	<LOD
7:3 FTCA	0.0050	0.021	0.011	0.0081	0.017
6:2 FTUCA	<LOD	<LOD	<LOD	<LOD	<LOD
8:2 FTUCA	n.a.	n.a.	n.a.	n.a.	n.a.
10:2 FTUCA	n.a.	n.a.	n.a.	n.a.	n.a.
4:2 FTSA	0.0047	0.0023	0.0058	0.0055	0.0073
6:2 FTSA	<LOD	<LOD	<LOD	<LOD	<LOD
8:2 FTSA	0.0047	0.0031	0.0022	<LOD	0.0078
10:2 FTSA	n.a.	n.a.	n.a.	n.a.	n.a.
PFHxPA	<LOD	<LOD	<LOD	<LOD	<LOD
PFOPA	n.a.	n.a.	n.a.	n.a.	n.a.
PFDPA	n.a.	n.a.	n.a.	n.a.	n.a.
6:6 PFPiA	<LOD	<LOD	<LOD	<LOD	<LOD
6:8 PFPiA	n.a.	n.a.	n.a.	n.a.	n.a.
8:8 PFPiA	n.a.	n.a.	n.a.	n.a.	n.a.
8:2 Cl-PFESA	<LOD	<LOD	<LOD	<LOD	<LOD
6:2 Cl-PFESA	n.a.	n.a.	n.a.	n.a.	n.a.
ADONA	<LOD	<LOD	<LOD	<LOD	<LOD
HFPO-DA (GenX)	n.a.	n.a.	n.a.	n.a.	n.a.

MeFOSAA	0.028	0.038	0.0052	0.0058	0.0048
EtFOSAA	0.0027	0.0092	<LOD	0.0035	<LOD
diSAmPAP	<LOD	<LOD	<LOD	<LOD	<LOD
6:2 monoPAP	n.a.	n.a.	n.a.	n.a.	n.a.
8:2 monoPAP	n.a.	n.a.	n.a.	n.a.	n.a.
10:2 monoPAP	n.a.	n.a.	n.a.	n.a.	n.a.
6:2 diPAP	<LOD	<LOD	0.038	<LOD	<LOD
8:2 diPAP	<LOD	<LOD	<LOD	<LOD	<LOD
6:2/8:2 diPAP	<LOD	<LOD	<LOD	<LOD	<LOD
10:2 diPAP	n.a.	n.a.	n.a.	n.a.	n.a.
PFOS-branch ∑	0.051	0.10	0.050	0.15	0.13

Table S9: PFAS concentrations of the placental pool tissue samples plaT-K to plaT-M in ng/g (not concentrated value, so the concentrations represents the mean of each single sample in the respective pool) – the number in the "()" represents the number of samples which were pooled together

**Summary of the concentrations [ng/g] of pooled placental tissue from K-M**			
**Compound**	plaT-K (3)	plaT-L (3)	plaT-M (2)
PFBA	<LOD	<LOD	0.032
PFPeA	<LOD	<LOD	<LOD
PFHxA	<LOD	<LOD	<LOD
PFHpA	<LOD	0.014	0.016
PFOA	0.36	0.37	0.55
PFNA	0.13	0.077	0.099
PFDA	0.082	0.034	0.053
PFUnDA	0.073	0.032	0.031
PFDoDA	0.028	0.010	0.018
PFTrDA	0.033	0.021	<LOD
PFTDA	0.034	0.019	0.055
PFHxDA	n.a.	n.a.	n.a.

PFOcDA	n.a.	n.a.	n.a.
PFBS	<LOD	<LOD	<LOD
PFPeS	<LOD	<LOD	<LOD
PFHxS	0.084	0.11	0.14
PFHpS	0.0065	0.010	0.013
L-PFOS	0.47	0.28	0.50
PFNS	n.a.	n.a.	n.a.
PFDS	<LOD	<LOD	<LOD
PFDoDS	<LOD	<LOD	<LOD
PFECHS	n.a.	n.a.	n.a.
FBSA	<LOD	<LOD	<LOD
MeFBSA	<LOD	<LOD	<LOD
FHxSA	<LOD	<LOD	<LOD
MeFHxSA	<LOD	<LOD	<LOD
FOSA	n.a.	n.a.	n.a.
MeFOSA	n.a.	n.a.	n.a.
EtFOSA	n.a.	n.a.	n.a.
MeFOSE	n.a.	n.a.	n.a.
EtFOSE	n.a.	n.a.	n.a.
3:3 FTCA	<LOD	<LOD	<LOD
5:3 FTCA	<LOD	<LOD	<LOD
7:3 FTCA	0.015	0.011	0.015
6:2 FTUCA	<LOD	<LOD	<LOD
8:2 FTUCA	n.a.	n.a.	n.a.
10:2 FTUCA	n.a.	n.a.	n.a.
4:2 FTSA	0.011	0.0055	0.0089
6:2 FTSA	<LOD	<LOD	<LOD
8:2 FTSA	<LOD	<LOD	<LOD
10:2 FTSA	n.a.	n.a.	n.a.
PFHxPA	<LOD	<LOD	<LOD
PFOPA	n.a.	n.a.	n.a.
PFDPA	n.a.	n.a.	n.a.

6:6 PFPiA	<LOD	<LOD	<LOD
6:8 PFPiA	n.a.	n.a.	n.a.
8:8 PFPiA	n.a.	n.a.	n.a.
8:2 Cl-PFESA	<LOD	<LOD	<LOD
6:2 Cl-PFESA	n.a.	n.a.	n.a.
ADONA	<LOD	<LOD	<LOD
HFPO-DA (GenX)	n.a.	n.a.	n.a.
MeFOSAA	0.0069	0.0050	0.012
EtFOSAA	0.011	0.0027	<LOD
diSAmPAP	<LOD	<LOD	<LOD
6:2 monoPAP	n.a.	n.a.	n.a.
8:2 monoPAP	n.a.	n.a.	n.a.
10:2 monoPAP	n.a.	n.a.	n.a.
6:2 diPAP	<LOD	<LOD	<LOD
8:2 diPAP	<LOD	<LOD	<LOD
6:2/8:2 diPAP	<LOD	<LOD	<LOD
10:2 diPAP	n.a.	n.a.	n.a.
PFOS-branch ∑	0.090	0.061	0.12

Table S10: PFAS concentrations of the cord serum pool samples cordS-B to cordS-H in ng/mL (not concentrated value, so the concentrations represents the mean of each single sample in the respective pool) – the number in the "()" represents the number of samples which were pooled together

**Summary of the concentrations [ng/mL] of pooled cord serum from B-H**					
**Compound**	cordS-B (2)	cordS-C (3)	cordS-D (3)	cordS-E (1)	cordS-H (3)
PFBA	<LOD	<LOD	<LOD	<LOD	<LOD
PFPeA	<LOD	<LOD	<LOD	<LOD	<LOD
PFHxA	<LOD	<LOD	<LOD	<LOD	0.011
PFHpA	0.045	0.014	0.026	<LOD	0.019
PFOA	1.40	0.72	0.84	0.093	0.81

PFNA	0.13	0.12	0.17	0.040	0.21
PFDA	0.047	0.037	0.040	<LOD	0.065
PFUnDA	0.020	0.017	0.025	0.039	0.037
PFDoDA	<LOD	0.010	0.010	<LOD	0.015
PFTrDA	n.a.	n.a.	n.a.	n.a.	n.a.
PFTDA	n.a.	n.a.	n.a.	n.a.	n.a.
PFHxDA	n.a.	n.a.	n.a.	n.a.	n.a.
PFOcDA	n.a.	n.a.	n.a.	n.a.	n.a.
PFBS	0.0076	0.0064	0.0020	<LOD	0.0044
PFPeS	0.0032	0.0064	0.0025	<LOD	0.0034
PFHxS	0.23	0.24	0.14	<LOQ	0.18
PFHpS	<LOD	0.017	0.010	<LOQ	0.017
L-PFOS	0.32	0.34	0.29	0.13	0.35
PFNS	<LOD	<LOD	<LOD	<LOD	<LOD
PFDS	<LOD	<LOD	<LOD	<LOD	<LOD
PFDoDS	n.a.	n.a.	n.a.	n.a.	n.a.
PFECHS	0.0019	0.0023	0.0027	<LOD	0.0075
FBSA	<LOD	<LOD	<LOD	<LOD	<LOD
MeFBSA	<LOD	<LOD	<LOD	<LOD	<LOD
FHxSA	<LOD	<LOD	<LOD	<LOD	<LOD
MeFHxSA	<LOD	<LOD	<LOD	<LOD	<LOD
FOSA	<LOD	<LOD	<LOD	<LOD	<LOD
MeFOSA	<LOD	<LOD	<LOD	<LOD	<LOD
EtFOSA	n.a.	n.a.	n.a.	n.a.	n.a.
MeFOSE	<LOD	<LOD	<LOD	<LOD	<LOD
EtFOSE	<LOD	<LOD	<LOD	<LOD	<LOD
3:3 FTCA	<LOD	<LOD	<LOD	<LOD	<LOD
5:3 FTCA	<LOD	<LOD	<LOD	<LOD	<LOD
7:3 FTCA	<LOQ	<LOD	<LOD	<LOD	<LOQ
6:2 FTUCA	<LOD	<LOD	<LOD	<LOD	<LOD
8:2 FTUCA	<LOD	<LOD	<LOD	<LOD	<LOD
10:2 FTUCA	<LOD	<LOD	<LOD	<LOD	<LOD

4:2 FTSA	<LOD	<LOD	<LOD	<LOD	<LOD
6:2 FTSA	<LOQ	<LOD	0.0070	<LOD	0.0072
8:2 FTSA	<LOD	<LOD	<LOD	<LOD	<LOD
10:2 FTSA	n.a.	n.a.	n.a.	n.a.	n.a.
PFHxPA	<LOD	<LOD	<LOD	<LOD	<LOD
PFOPA	n.a.	n.a.	n.a.	n.a.	n.a.
PFDPA	n.a.	n.a.	n.a.	n.a.	n.a.
6:6 PFPiA	<LOD	<LOD	<LOD	<LOD	<LOD
6:8 PFPiA	n.a.	n.a.	n.a.	n.a.	n.a.
8:8 PFPiA	n.a.	n.a.	n.a.	n.a.	n.a.
8:2 Cl-PFESA	<LOD	<LOD	<LOD	<LOD	<LOD
6:2 Cl-PFESA	<LOD	<LOD	<LOD	<LOD	<LOD
ADONA	0.014	0.0076	<LOD	<LOD	<LOD
HFPO-DA (GenX)	n.a.	n.a.	n.a.	n.a.	n.a.
MeFOSAA	<LOD	<LOQ	0.011	<LOD	0.022
EtFOSAA	<LOD	<LOD	<LOD	<LOD	<LOQ
diSAmPAP	n.a.	n.a.	n.a.	n.a.	n.a.
6:2 monoPAP	n.a.	n.a.	n.a.	n.a.	n.a.
8:2 monoPAP	n.a.	n.a.	n.a.	n.a.	n.a.
10:2 monoPAP	<LOD	<LOD	<LOD	<LOD	<LOD
6:2 diPAP	<LOD	<LOD	<LOD	<LOD	<LOD
8:2 diPAP	n.a.	n.a.	n.a.	n.a.	n.a.
6:2/8:2 diPAP	n.a.	n.a.	n.a.	n.a.	n.a.
10:2 diPAP	n.a.	n.a.	n.a.	n.a.	n.a.
PFOS-branch ∑	0.18	0.17	0.17	0.011	0.14

Table S11: PFAS concentrations of the cord serum pool samples cordS-K to cordS-U in ng/mL (not concentrated value, so the concentrations represents the mean of each single sample in the respective pool) – the number in the “()” represents the number of samples which were pooled together

**Summary of the concentrations [ng/mL] of pooled cord serum from K-U**						
**Compound**	cordS-K (3)	cordS-L (3)	cordS-N (3)	cordS-O (2)	cordS-S (3)	cordS-U (2)
PFBA	<LOD	<LOD	<LOD	<LOD	<LOD	<LOD
PFPeA	<LOD	<LOD	<LOD	<LOD	<LOD	<LOD
PFHxA	0.027	<LOD	<LOD	<LOD	<LOD	<LOD
PFHpA	0.032	0.021	0.070	0.061	0.022	0.0065
PFOA	1.42	0.75	0.86	1.08	0.89	0.54
PFNA	0.29	0.084	0.11	0.23	0.13	0.098
PFDA	0.090	0.026	0.038	0.067	0.040	0.046
PFUnDA	0.073	0.026	0.021	0.047	0.027	0.046
PFDoDA	0.013	<LOD	<LOD	<LOD	<LOQ	<LOQ
PFTrDA	n.a.	n.a.	n.a.	n.a.	n.a.	n.a.
PFTDA	n.a.	n.a.	n.a.	n.a.	n.a.	n.a.
PFHxDA	n.a.	n.a.	n.a.	n.a.	n.a.	n.a.
PFOcDA	n.a.	n.a.	n.a.	n.a.	n.a.	n.a.
PFBS	0.0084	0.0060	0.0099	<LOD	0.0094	0.0046
PFPeS	0.0081	0.0050	0.0053	0.0054	0.0023	<LOD
PFHxS	0.34	0.26	0.20	0.27	0.23	0.28
PFHpS	0.026	0.019	0.014	0.037	0.012	0.020
L-PFOS	0.62	0.21	0.31	0.84	0.34	0.47
PFNS	<LOD	<LOD	<LOD	<LOD	<LOD	<LOD
PFDS	<LOD	<LOD	<LOD	<LOD	<LOD	<LOD
PFDoDS	n.a.	n.a.	n.a.	n.a.	n.a.	n.a.
PFECHS	0.0027	0.0032	0.0016	0.0022	0.0019	< LOQ
FBSA	<LOD	<LOD	<LOD	<LOD	<LOD	<LOD
MeFBSA	<LOD	<LOD	<LOD	<LOD	<LOD	<LOD
FHxSA	<LOD	<LOD	<LOD	<LOD	<LOD	<LOD
MeFHxSA	<LOD	<LOD	<LOD	<LOD	<LOD	<LOD

FOSA	<LOD	<LOD	<LOD	<LOD	<LOD	<LOD
MeFOSA	<LOD	<LOD	<LOD	<LOD	<LOD	<LOD
EtFOSA	n.a.	n.a.	n.a.	n.a.	n.a.	n.a.
MeFOSE	<LOD	<LOD	<LOD	<LOD	<LOD	<LOD
EtFOSE	<LOD	<LOD	<LOD	<LOD	<LOD	<LOD
3:3 FTCA	<LOD	<LOD	<LOD	<LOD	<LOD	<LOD
5:3 FTCA	<LOD	<LOD	<LOD	<LOD	<LOD	<LOD
7:3 FTCA	0.0050	<LOD	<LOD	<LOD	<LOQ	<LOD
6:2 FTUCA	<LOD	<LOD	<LOD	<LOD	<LOD	<LOD
8:2 FTUCA	<LOD	<LOD	<LOD	<LOD	<LOD	<LOD
10:2 FTUCA	<LOD	<LOD	<LOD	<LOD	<LOD	<LOD
4:2 FTSA	<LOD	<LOD	<LOD	<LOD	<LOD	<LOD
6:2 FTSA	0.013	0.0072	0.0045	0.010	0.0070	0.0065
8:2 FTSA	<LOD	<LOD	<LOD	<LOD	<LOD	<LOD
10:2 FTSA	n.a.	n.a.	n.a.	n.a.	n.a.	n.a.
PFHxPA	<LOD	<LOD	<LOD	<LOD	<LOD	<LOD
PFOPA	n.a.	n.a.	n.a.	n.a.	n.a.	n.a.
PFDPA	n.a.	n.a.	n.a.	n.a.	n.a.	n.a.
6:6 PFPiA	<LOD	<LOD	<LOD	<LOD	<LOD	<LOD
6:8 PFPiA	n.a.	n.a.	n.a.	n.a.	n.a.	n.a.
8:8 PFPiA	n.a.	n.a.	n.a.	n.a.	n.a.	n.a.
8:2 Cl-PFESA	<LOD	<LOD	<LOD	<LOD	<LOD	<LOD
6:2 Cl-PFESA	<LOD	<LOD	<LOD	<LOD	<LOD	<LOD
ADONA	0.021	0.027	0.027	0.0094	0.0039	0.0053
HFPO-DA (GenX)	n.a.	n.a.	n.a.	n.a.	n.a.	n.a.
MeFOSAA	<LOD	<LOD	0.0051	0.016	<LOD	<LOD
EtFOSAA	<LOD	<LOD	<LOD	0.17	<LOD	<LOD
diSAmPAP	n.a.	n.a.	n.a.	n.a.	n.a.	n.a.
6:2 monoPAP	n.a.	n.a.	n.a.	n.a.	n.a.	n.a.
8:2 monoPAP	n.a.	n.a.	n.a.	n.a.	n.a.	n.a.
10:2 monoPAP	<LOD	<LOD	<LOD	<LOD	<LOD	<LOD
6:2 diPAP	<LOD	<LOD	<LOD	<LOD	<LOD	<LOD

8:2 diPAP	n.a.	n.a.	n.a.	n.a.	n.a.	n.a.
6:2/8:2 diPAP	n.a.	n.a.	n.a.	n.a.	n.a.	n.a.
10:2 diPAP	n.a.	n.a.	n.a.	n.a.	n.a.	n.a.
PFOS-branch ∑	0.31	0.15	0.14	0.29	0.15	0.25

Table S12: Blanks (BW): for BW 1 and BW 2 3 mL Milli Q Water (MQ) were used, for BW 3 2 mL bovine serum (b.s.) was used, for BW 4 bovine serum spiked with inorganic fluorine (inorg. F), and for BW 5 2 mL bovine serum spiked with inorganic fluorine but without the additional washing step with 18 mL 0.01% ammonium solution in MQ (no add w.).

**Concentrations of the blanks 1-5 for the pooled serum samples**					
**Compound**	BW 1 (3 mL MQ)	BW 2 (3 mL MQ)	BW 3 (2 mL b.s.)	BW 4 (inorg. F)	BW 5 (no add w.)
PFBA	<LOD	<LOD	<LOD	<LOD	<LOD
PFPeA	<LOD	<LOD	<LOD	<LOD	<LOD
PFHxA	0.0054	0.0087	0.0091	0.0096	<LOD
PFHpA	<LOD	<LOD	<LOD	<LOD	<LOD
PFOA	0.0042	0.0068	0.0086	0.0075	<LOD
PFNA	<LOD	<LOD	0.028	0.025	0.017
PFDA	<LOD	<LOQ	<LOQ	<LOQ	<LOQ
PFUnDA	0.010	0.013	0.0099	<LOQ	<LOD
PFDoDA	0.0017	0.0017	0.0026	0.0026	0.0026
PFTrDA	n.a.	n.a.	n.a.	n.a.	n.a.
PFTDA	n.a.	n.a.	n.a.	n.a.	n.a.
PFHxDA	n.a.	n.a.	n.a.	n.a.	n.a.
PFOcDA	n.a.	n.a.	n.a.	n.a.	n.a.
PFBS	0.0012	0.0007	<LOD	<LOD	<LOD
PFPeS	<LOD	<LOD	<LOD	<LOQ	0.0015
PFHxS	<LOQ	0.0046	0.041	0.049	0.040
PFHpS	<LOD	<LOD	0.0021	0.0050	0.0073
L-PFOS	<LOD	<LOD	0.065	0.073	0.072
PFNS	<LOD	<LOD	<LOD	<LOD	<LOD
PFDS	<LOD	<LOD	<LOD	<LOD	<LOD
PFDoDS	n.a.	n.a.	n.a.	n.a.	n.a.

PFECHS	0.0002	0.0002	0.0003	0.0003	0.0003
FBSA	<LOD	<LOD	<LOD	<LOD	<LOD
MeFBSA	n.a.	n.a.	n.a.	n.a.	n.a.
FHxSA	<LOD	<LOD	0.0229	<LOD	<LOD
MeFHxSA	<LOD	<LOD	<LOD	<LOD	<LOD
FOSA	<LOD	<LOD	<LOD	<LOD	<LOD
MeFOSA	<LOD	<LOD	<LOD	<LOD	<LOD
EtFOSA	<LOD	<LOD	<LOD	<LOD	<LOD
MeFOSE	<LOD	<LOD	<LOD	<LOD	<LOD
EtFOSE	<LOD	<LOD	<LOD	<LOD	<LOD
3:3 FTCA	<LOD	<LOD	<LOD	<LOD	<LOD
5:3 FTCA	<LOD	<LOD	<LOD	<LOD	<LOD
7:3 FTCA	<LOD	<LOD	<LOD	<LOD	<LOD
6:2 FTUCA	<LOD	<LOD	<LOD	<LOD	<LOD
8:2 FTUCA	<LOD	<LOD	<LOD	<LOD	<LOD
10:2 FTUCA	<LOD	<LOD	<LOD	<LOD	<LOD
4:2 FTSA	<LOD	<LOD	<LOD	<LOD	<LOD
6:2 FTSA	n.a.	n.a.	n.a.	n.a.	n.a.
8:2 FTSA	<LOD	<LOD	<LOD	<LOD	<LOD
10:2 FTSA	<LOD	<LOD	<LOD	<LOD	<LOD
PFHxPA	<LOD	<LOD	<LOD	<LOD	<LOD
PFOPA	n.a.	n.a.	n.a.	n.a.	n.a.
PFDPA	n.a.	n.a.	n.a.	n.a.	n.a.
6:6 PFPiA	<LOD	<LOD	<LOD	<LOD	<LOD
6:8 PFPiA	n.a.	n.a.	n.a.	n.a.	n.a.
8:8 PFPiA	n.a.	n.a.	n.a.	n.a.	n.a.
8:2 Cl-PFESA	<LOD	<LOD	<LOD	<LOD	<LOD
6:2 Cl-PFESA	<LOD	<LOD	<LOD	<LOD	<LOD
ADONA	<LOD	<LOD	<LOD	<LOD	<LOD
HFPO-DA (GenX)	n.a.	n.a.	n.a.	n.a.	n.a.
MeFOSAA	<LOD	<LOD	<LOD	<LOD	<LOD
EtFOSAA	<LOD	<LOD	<LOD	<LOD	<LOD

diSAmPAP	n.a.	n.a.	n.a.	n.a.	n.a.
6:2 monoPAP	n.a.	n.a.	n.a.	n.a.	n.a.
8:2 monoPAP	n.a.	n.a.	n.a.	n.a.	n.a.
10:2 monoPAP	<LOD	<LOD	<LOD	<LOD	<LOD
6:2 diPAP	<LOD	<LOD	<LOD	<LOD	<LOD
8:2 diPAP	n.a.	n.a.	n.a.	n.a.	n.a.
6:2/8:2 diPAP	n.a.	n.a.	n.a.	n.a.	n.a.
10:2 diPAP	n.a.	n.a.	n.a.	n.a.	n.a.
PFOS-branch ∑	<LOD	<LOD	0.023	0.020	0.024

Table S13: Blanks (BW): for BW 6 and BW 7 3 mL Milli Q Water (MQ) were used, for BW 8 2 mL bovine serum (b.s.) was used, for BW 9 bovine serum spiked with inorganic fluorine (inorg. F), and for BW 10 2 mL bovine serum spiked with inorganic fluorine but without the additional washing step with 18 mL 0.01% ammonium solution in MQ (no add w.).

**Concentrations of the blanks 6-10 for the pooled serum samples**					
**Compound**	BW 6 (3 mL MQ)	BW 7 (3 mL MQ)	BW 8 (2 mL b.s.)	BW 9 (inorg. F)	BW 10 (no add w.)
PFBA	<LOD	<LOD	<LOD	<LOD	0.21
PFPeA	<LOD	<LOD	<LOD	<LOD	<LOD
PFHxA	0.015	0.019	0.023	0.021	<LOD
PFHpA	<LOD	<LOD	<LOQ	<LOD	<LOD
PFOA	0.012	0.013	0.016	0.015	<LOQ
PFNA	<LOD	<LOD	0.032	0.031	0.011
PFDA	<LOD	<LOD	0.0215	0.024	<LOQ
PFUnDA	0.0052	0.011	0.017	0.011	0.013
PFDoDA	0.0017	0.0017	0.0026	0.0026	0.0026
PFTrDA	n.a.	n.a.	n.a.	n.a.	n.a.
PFTDA	n.a.	n.a.	n.a.	n.a.	n.a.
PFHxDA	n.a.	n.a.	n.a.	n.a.	n.a.
PFOcDA	n.a.	n.a.	n.a.	n.a.	n.a.
PFBS	<LOD	<LOD	<LOD	<LOD	<LOD

PFPeS	<LOD	<LOD	<LOD	<LOD	<LOD
PFHxS	0.0083	0.012	0.084	0.062	0.061
PFHpS	<LOD	<LOD	<LOD	0.0038	0.0048
L-PFOS	<LOD	<LOD	0.10	0.11	0.11
PFNS	<LOD	<LOD	<LOD	<LOD	<LOD
PFDS	<LOD	<LOD	<LOD	<LOD	<LOD
PFDoDS	n.a.	n.a.	n.a.	n.a.	n.a.
PFECHS	0.0002	0.0002	0.0003	0.0003	0.0003
FBSA	<LOD	<LOD	<LOD	<LOD	<LOD
MeFBSA	<LOD	<LOD	<LOD	<LOD	<LOD
PFHxSA	<LOD	<LOD	<LOD	0.039	<LOD
MePFHxSA	<LOD	<LOD	<LOD	<LOD	<LOD
PFOSA	<LOD	<LOD	<LOD	<LOD	<LOD
MeFOSA	<LOD	<LOD	<LOD	<LOD	<LOD
EtFOSA	n.a.	n.a.	n.a.	n.a.	n.a.
MeFOSE	<LOD	<LOD	<LOD	<LOD	<LOD
EtFOSE	<LOD	<LOD	<LOD	<LOD	<LOD
3:3 FTCA	<LOD	<LOD	<LOD	<LOD	<LOD
5:3 FTCA	<LOD	<LOD	<LOD	<LOD	<LOD
7:3 FTCA	<LOD	<LOD	<LOD	<LOD	<LOD
6:2 FTUCA	<LOD	<LOD	<LOD	<LOD	<LOD
8:2 FTUCA	<LOD	<LOD	<LOD	<LOD	<LOD
10:2 FTUCA	<LOD	<LOD	<LOD	<LOD	<LOD
4:2 FTSA	<LOD	<LOD	<LOD	<LOD	<LOD
6:2 FTSA	0.0058	0.010	0.63	0.61	0.59
8:2 FTSA	<LOD	<LOD	<LOD	<LOD	<LOD
10:2 FTSA	n.a.	n.a.	n.a.	n.a.	n.a.
PFHxPA	<LOD	<LOD	<LOD	<LOD	<LOD
PFOPA	n.a.	n.a.	n.a.	n.a.	n.a.
PFDPA	n.a.	n.a.	n.a.	n.a.	n.a.
6:6 PFPiA	<LOD	<LOD	<LOD	<LOD	<LOD
6:8 PFPiA	n.a.	n.a.	n.a.	n.a.	n.a.

8:8 PFPiA	n.a.	n.a.	n.a.	n.a.	n.a.
8:2 Cl-PFESA	<LOD	<LOD	<LOD	<LOD	<LOD
6:2 Cl-PFESA	<LOD	<LOD	<LOD	<LOD	<LOD
ADONA	<LOD	<LOD	0.0030	<LOD	<LOD
HFPO-DA (GenX)	n.a.	n.a.	n.a.	n.a.	n.a.
N-MeFOSAA	<LOD	<LOD	<LOD	<LOQ	<LOD
N-EtFOSAA	<LOD	<LOD	<LOD	<LOD	<LOD
diSAmPAP	n.a.	n.a.	n.a.	n.a.	n.a.
6:2 monoPAP	n.a.	n.a.	n.a.	n.a.	n.a.
8:2 monoPAP	n.a.	n.a.	n.a.	n.a.	n.a.
10:2 monoPAP	<LOD	<LOD	<LOD	<LOD	<LOD
6:2 diPAP	<LOD	<LOD	<LOD	<LOD	<LOD
8:2 diPAP	n.a.	n.a.	n.a.	n.a.	n.a.
6:2/8:2 diPAP	n.a.	n.a.	n.a.	n.a.	n.a.
10:2 diPAP	n.a.	n.a.	n.a.	n.a.	n.a.
PFOS-branch ∑	0.0013	0.0003	0.026	0.033	0.047

Table S14: Blanks (BW): for BW 11 and BW 12 3 mL Milli Q Water (MQ) were used, for BW 13 2 mL bovine serum (b.s.) was used, for BW 14 and BW 15 bovine serum spiked with inorganic fluorine (inorg. F), and for BW 16 2 mL bovine serum spiked with inorganic fluorine but without the additional washing step with 18 mL 0.01% ammonium solution in MQ (no add w.).

**Concentrations of the blanks 11-16 for the pooled serum samples**						
**Compound**	BW 11 (3 mL MQ)	BW 12 (3 mL MQ)	BW 13 (2 mL b.s.)	BW 14 (inorg. F)	BW 15 (inorg. F)	BW 16 (no add w.)
PFBA	<LOD	<LOD	<LOD	<LOD	<LOD	0.0571
PFPeA	<LOD	<LOD	<LOD	<LOD	<LOD	<LOD
PFHxA	<LOD	<LOD	<LOD	<LOD	<LOD	<LOD
PFHpA	<LOQ	<LOD	<LOD	<LOD	<LOD	<LOD
PFOA	0.013	0.014	0.021	0.021	0.042	0.017
PFNA	<LOD	<LOD	0.021	0.023	0.028	0.010
PFDA	<LOD	<LOD	0.018	<LOQ	0.023	<LOQ

PFUnDA	0.0050	0.0063	0.0095	0.017	0.017	0.0091
PFDoDA	0.0017	0.0017	0.0026	0.0026	0.0026	<LOD
PFTrDA	n.a.	n.a.	n.a.	n.a.	n.a.	n.a.
PFTDA	n.a.	n.a.	n.a.	n.a.	n.a.	n.a.
PFHxDA	n.a.	n.a.	n.a.	n.a.	n.a.	n.a.
PFOcDA	n.a.	n.a.	n.a.	n.a.	n.a.	n.a.
PFBS	<LOD	<LOD	<LOD	<LOD	<LOD	<LOD
PFPeS	<LOD	<LOD	<LOD	<LOD	<LOD	<LOD
PFHxS	0.0065	0.0096	0.049	0.047	0.11	0.034
PFHpS	<LOD	<LOD	0.0040	0.0045	0.0010	0.0013
L-PFOS	<LOD	<LOD	0.066	0.086	0.070	0.049
PFNS	<LOD	<LOD	<LOD	<LOD	<LOD	<LOD
PFDS	<LOD	<LOD	<LOD	<LOD	<LOD	<LOD
PFDoDS	n.a.	n.a.	n.a.	n.a.	n.a.	n.a.
PFECHS	0.0002	0.0002	0.0003	0.0003	0.0003	0.0035
FBSA	<LOD	<LOD	<LOD	<LOD	<LOD	<LOD
MeFBSA	<LOD	<LOD	<LOD	<LOD	<LOD	<LOD
FHxSA	<LOD	<LOD	<LOD	<LOD	<LOD	<LOD
MeFHxSA	<LOD	<LOD	<LOD	<LOD	<LOD	<LOD
FOSA	<LOD	<LOD	<LOD	<LOD	<LOD	<LOD
MeFOSA	<LOD	<LOD	<LOD	<LOD	<LOD	<LOD
EtFOSA	n.a.	n.a.	n.a.	n.a.	n.a.	n.a.
MeFOSE	<LOD	<LOD	<LOD	<LOD	<LOD	<LOD
EtFOSE	<LOD	<LOD	<LOD	<LOD	<LOD	<LOD
3:3 FTCA	<LOD	<LOD	<LOD	<LOD	<LOD	<LOD
5:3 FTCA	<LOD	<LOD	<LOD	<LOD	<LOD	<LOD
7:3 FTCA	<LOD	<LOD	<LOD	<LOD	<LOD	<LOD
6:2 FTUCA	<LOD	<LOD	<LOD	<LOD	<LOD	<LOD
8:2 FTUCA	<LOD	<LOD	<LOD	<LOD	<LOD	<LOD
10:2 FTUCA	<LOD	<LOD	<LOD	<LOD	<LOD	<LOD
4:2 FTSA	<LOD	<LOD	<LOD	<LOD	<LOD	<LOD
6:2 FTSA	0.0045	0.0045	0.40	0.39	0.46	0.36

8:2 FTSA	<LOD	<LOD	<LOD	<LOD	<LOD	<LOD
10:2 FTSA	n.a.	n.a.	n.a.	n.a.	n.a.	n.a.
PFHxPA	<LOD	<LOD	<LOD	<LOD	<LOD	<LOD
PFOPA	n.a.	n.a.	n.a.	n.a.	n.a.	n.a.
PFDPA	n.a.	n.a.	n.a.	n.a.	n.a.	n.a.
6:6 PFPiA	<LOD	<LOD	<LOD	<LOD	<LOD	<LOD
6:8 PFPiA	n.a.	n.a.	n.a.	n.a.	n.a.	n.a.
8:8 PFPiA	n.a.	n.a.	n.a.	n.a.	n.a.	n.a.
8:2 Cl-PFESA	<LOD	<LOD	<LOD	<LOD	<LOD	<LOD
6:2 Cl-PFESA	<LOD	<LOD	<LOD	<LOD	<LOD	<LOD
ADONA	<LOD	0.0015	<LOD	<LOD	<LOD	<LOD
HFPO-DA (GenX)	n.a.	n.a.	n.a.	n.a.	n.a.	n.a.
MeFOSAA	<LOD	<LOD	<LOD	<LOD	<LOD	<LOD
EtFOSAA	<LOD	<LOD	<LOD	<LOD	<LOD	<LOD
diSAmPAP	n.a.	n.a.	n.a.	n.a.	n.a.	n.a.
6:2 monoPAP	n.a.	n.a.	n.a.	n.a.	n.a.	n.a.
8:2 monoPAP	n.a.	n.a.	n.a.	n.a.	n.a.	n.a.
10:2 monoPAP	n.n.	n.n.	n.n.	n.n.	n.n.	n.n.
6:2 diPAP	n.n.	n.n.	n.n.	n.n.	n.n.	n.n.
8:2 diPAP	n.a.	n.a.	n.a.	n.a.	n.a.	n.a.
6:2/8:2 diPAP	n.a.	n.a.	n.a.	n.a.	n.a.	n.a.
10:2 diPAP	n.a.	n.a.	n.a.	n.a.	n.a.	n.a.
PFOS-branch ∑	0.0000	0.0028	0.030	0.024	0.015	0.013

Table S15: Blanks (BW): for BW 1 and BW 3 3 mL Milli Q Water (MQ) were used spiked with inorganic fluorine (inorg. F), and for BW 2 and BW 4 3 mL MQ were used without the addition of inorganic fluorine.

**Concentrations of the blanks 1-4 for the pooled placental tissue samples**				
**Compound**	BW 1<br>(3 mL MQ, inorg. F)	BW 2<br>(no inorg. F)	BW 3<br>(3 mL MQ, inorg. F)	BW 4<br>(no inorg. F)
PFBA	<LOQ	<LOQ	<LOD	<LOD

PFPeA	<LOD	<LOD	<LOD	<LOD
PFHxA	0.012	0.011	0.014	0.0077
PFHpA	<LOQ	<LOD	<LOD	<LOD
PFOA	0.011	0.010	0.016	0.016
PFNA	0.0026	0.0020	0.0043	0.0057
PFDA	<LOD	<LOQ	<LOQ	<LOQ
PFUnDA	0.016	0.015	0.015	0.020
PFDoDA	<LOD	<LOD	<LOD	<LOD
PFTrDA	<LOD	<LOD	<LOD	<LOD
PFTDA	<LOD	<LOD	<LOD	<LOD
PFHxDA	n.a.	n.a.	n.a.	n.a.
PFOcDA	n.a.	n.a.	n.a.	n.a.
PFBS	<LOD	<LOD	<LOD	<LOD
PFPeS	<LOD	<LOD	<LOD	<LOD
PFHxS	0.0083	0.0076	0.012	0.016
PFHpS	<LOD	<LOD	<LOD	<LOD
L-PFOS	<LOD	<LOD	<LOD	<LOD
PFNS	n.a.	n.a.	n.a.	n.a.
PFDS	<LOD	<LOD	<LOD	<LOD
PFDoDS	<LOD	<LOD	<LOD	<LOD
PFECHS	n.a.	n.a.	n.a.	n.a.
FBSA	<LOD	<LOD	<LOD	<LOD
MeFBSA	<LOD	<LOD	<LOD	<LOD
FHxSA	<LOD	<LOD	<LOD	<LOD
MeFHxSA	<LOD	<LOD	<LOD	<LOD
FOSA	n.a.	n.a.	n.a.	n.a.
MeFOSA	n.a.	n.a.	n.a.	n.a.
EtFOSA	n.a.	n.a.	n.a.	n.a.
MeFOSE	n.a.	n.a.	n.a.	n.a.
EtFOSE	n.a.	n.a.	n.a.	n.a.
3:3 FTCA	<LOD	<LOD	<LOD	<LOD
5:3 FTCA	<LOD	<LOD	<LOD	<LOD

7:3 FTCA	<LOD	<LOD	<LOD	<LOD
6:2 FTUCA	<LOD	<LOD	<LOD	<LOD
8:2 FTUCA	n.a.	n.a.	n.a.	n.a.
10:2 FTUCA	n.a.	n.a.	n.a.	n.a.
4:2 FTSA	<LOD	<LOD	<LOD	<LOD
6:2 FTSA	<LOD	<LOD	<LOD	<LOD
8:2 FTSA	<LOD	<LOD	<LOD	<LOD
10:2 FTSA	n.a.	n.a.	n.a.	n.a.
PFHxPA	<LOD	<LOD	<LOD	<LOD
PFOPA	n.a.	n.a.	n.a.	n.a.
PFDPA	n.a.	n.a.	n.a.	n.a.
6:6 PFPiA	<LOD	<LOD	<LOD	<LOD
6:8 PFPiA	n.a.	n.a.	n.a.	n.a.
8:8 PFPiA	n.a.	n.a.	n.a.	n.a.
8:2 Cl-PFESA	<LOD	<LOD	<LOD	<LOD
6:2 Cl-PFESA	n.a.	n.a.	n.a.	n.a.
ADONA	0.017	0.046	0.017	0.046
HFPO-DA (GenX)	n.a.	n.a.	n.a.	n.a.
MeFOSAA	<LOD	<LOD	<LOD	<LOD
EtFOSAA	<LOD	<LOD	<LOD	<LOD
diSAmPAP	<LOD	<LOD	<LOD	<LOD
6:2 monoPAP	n.a.	n.a.	n.a.	n.a.
8:2 monoPAP	n.a.	n.a.	n.a.	n.a.
10:2 monoPAP	n.a.	n.a.	n.a.	n.a.
6:2 diPAP	<LOD	<LOD	<LOD	<LOD
8:2 diPAP	<LOD	<LOD	<LOD	<LOD
6:2/8:2 diPAP	<LOD	<LOD	<LOD	<LOD
10:2 diPAP	n.a.	n.a.	n.a.	n.a.
PFOS-branch ∑	<LOD	<LOD	<LOD	<LOD

Extractable organofluorine (EOF) concentrations of the samples

The extractable organofluorine concentrations for all sample pools are provided in Table S16 to Table S18. Table S19 provides detailed information on the sampel pooling and Table S20 shows extractable organofluorine concentrations from other studies compared to the present one.

Maternal serum (matS).

Table S16: Identified EOF in ng F/mL in maternal serum (matS). The LOQ was 2.7 ng F/mL except for the samples matS-B, matS-C, matS-E to matS-O, and matS-U, where the background noise inbetween the samples was higher and therefore no values of the CIC for those samples are available.

Sample name	CIC-EOF [ng F/mL]	Targeted [ng F/mL]	Identified [%]
matS-A	2.85	3.11	109.07
matS-B	<LOQ	2.74	
matS-C	<LOQ	3.16	
matS-D	2.90	2.53	87.12
matS-E	<LOQ	5.87	
matS-F	<LOQ	1.78	
matS-G	<LOQ	2.19	
matS-H	<LOQ	3.10	
matS-I	<LOQ	4.70	
matS-J	<LOQ	6.24	
matS-K	<LOQ	3.21	
matS-L	<LOQ	2.50	
matS-M	<LOQ	3.34	
matS-N	<LOQ	2.71	
matS-O	<LOQ	6.88	
matS-P	3.59	2.33	64.97
matS-Q	<LOQ	2.08	
matS-R	7.17	2.77	38.68
matS-S	2.99	2.08	69.50

matS-T	3.46	3.10	89.46
matS-U	<LOQ	3.47	

Placental tissue (plaT).

Table S17: Identified EOF in ng F/mL in placental tissue (plaT). The LOQ was 0.92 ng F/mL, except for plaT-I and plaT-J where the background noise inbetween the samples was higher and therefore no values of the CIC for those samples are available.

**Sample name**	**CIC-EOF [ng F/g]**	**Targeted [ng F/g]**	**Identified [%]**
plaT-A	1.43	0.57	39.43
plaT-B	1.08	0.53	49.13
plaT-C	1.07	0.53	49.40
plaT-D	1.85	0.77	41.49
plaT-E	<LOQ	0.88	
plaT-F	<LOQ	0.34	
plaT-G	<LOQ	0.78	
plaT-H	1.02	0.58	57.32
plaT-I	<LOQ	0.92	
plaT-J	<LOQ	1.36	
plaT-K	1.08	0.62	57.64
plaT-L	<LOQ	0.45	
plaT-M	<LOQ	0.74	

Cord serum (cordS).

Table S18: Identified EOF in ng F/mL in cord serum (cordS). The LOQ was 1.1 ng F/mL, except for cord-B and cord-S where the background noise inbetween the samples was higher and therefore no values of the CIC for those samples are available.

**Sample name**	**EOF-CIC [ng F/mL]**	**Targeted [ng F/mL]**	**Identified [%]**
cordS-B	<LOQ	1.77	
cordS-C	1.80	1.20	66.65
cordS-D	1.20	1.18	97.90
cordS-E	<LOQ	0.33	

cordS-H	1.36	1.27	93.53
cordS-K	2.10	2.23	106.28
cordS-L	<LOQ	1.06	
cordS-N	1.20	1.23	101.99
cordS-O	2.03	2.11	104.12
cordS-S	<LOQ	1.26	
cordS-U	1.80	1.24	69.04

Table S19: Detail information on the sample pooling – sample pools for which the EOF concentration was <LOQ are marked with a "*" and "n.a." was used if a sample pool was not available (e.g. not enough sample volume available for pooling)

Sample	Quantity of samples for pooling (sera; placenta)	matS [µL]	plaT [g]	cordS [µL]	Gender (child)	City	Weight [g]	Classification	Health
A	3 x 600 µL; 3 x 1.5 g	1800	4.5	n.a.	female	St. Pölten (STP)	≤2850	SGA	ok
B	2 x 600 µL (2); 2 x 3 g	1200	6	1200	female	Tulln (TU)	>2850	AGA	ok
C	3 x 600 µL (2); 3 x 2g	1800	6	1800	female	Tulln	>2850	AGA	ok
D	3 x 600 µL (2); 3 x 1.5 g	1800	4.5	1800	female	Tulln	>2850	AGA	ok
E	1 x 600 µL (2); 1 x 6 g	600	6*	600*	female	St. Pölten	>2850	AGA	ok
F	4 x 600 µL; 4 x 1.5 g	2400	6	n.a.	female	Tulln	>2850	AGA	ok
G	2 x 600 µL; 2 x 3 g	1200	6	n.a.	male	St. Pölten	>2850	AGA	PE
H	3 x 600 µL (2); 3 x 1.5 g	1800	4.5	1800	male	St Pölten	≤2850	SGA	ok
I	1 x 600 µL; 1 x 4.5 g	600	4.5*	n.a.	male	St. Pölten	≤2850	AGA	ok
J	2 x 600 µL; 2 x 2.25 g	1200	4.5	n.a.	male	Tulln	>2850	AGA	ok
K	3 x 600 µL (2); 3 x 1.5 g	1800	4.5	1800	male	Tulln	>2850	AGA	ok
L	3 x 600 µL (2); 3 x 1.5 g	1800	4.5*	1200*	male	Tulln	>2850	AGA	ok
M	2 x 600 µL; 2 x 1.5 g	1200	3	n.a.	male	Tulln	>2850	AGA	ok
N	3 x 600 µL (2)	1800	n.a.	1800	female	Tulln	>2850	AGA	GDM
O	2 x 600 µL (2)	1200	n.a.	1200	female	Tulln	mix	AGA	FG
P	3 x 600 µL	1800	n.a.	n.a.	female	Wien / STP	mix	AGA	FG
Q	3 x 600 µL	1800	n.a.	n.a.	female	Wien	>2850	AGA	ok
R	3 x 600 µL	1800	n.a.	n.a.	female	Wien	>2850	AGA	ok
S	3 x 600 µL (2)	1800	n.a.	1800*	male	Tulln	>2850	AGA	GDM

T	3 x 600 µL	1800	n.a.	n.a.	male	Wien / STP / TU	mix	AGA	FG
U	2 x 600 µL (2)	1200	n.a.	1200	male	Wien / TU	>2850	LGA	ok

Table S20: The comparison of EOF concentrations in ng F/mL of the present study with previous studies

Study	Year of sampling	Country	matS (or others) [ng F/mL]	plaT [ng F/g]	cordS [ng F/mL]
Present study	2019-2020	Austria	2.85 – 7.17	1.02 – 1.85	1.2 – 2.10
Yeung and Mabury (2016)[1]	2004	China	8.22 – 94.4 (whole blood, male and female)		
Yeung and Mabury (2016)[1]	1982-2009	Germany	9.42 – 42.5 (plasma, male and female)		
Miaz et al. (2020)[2]	1996-2017	Sweden	8.1 – 32.0 ng F/ g (serum, females)		
Yeung et al. (2008)[3]	2004	China	< 6 – 43.4 (whole blood, male and female)		
Miyake et al., (2007)[4]	2003-2004	Japan	< 6 – 8.9 (whole blood and serum, males)		
Miyake et al., (2007)[4]	2001	USA	17.8 – 59 (plasma, males)		

Correlation analyses and scatter plots

Figures S2 to S7 show the scatter plots for the total EOF levels (EOF-CIC) determined on the CIC and individual target compound levels for matS and plaT converted to their fluorine equivalents. Additionally, scatter plots are shown for unidentified EOF levels (EOF-unid) and individual target compounds. For the scatter plots, the results from the concentrated samples were used, however, the outcome after adjusting to the orginial sample pool volumes is equal. The figures also show the Spearmen correlation coefficienties (r_S) and the p-values. The grey zone represents the 95% confidence interval.

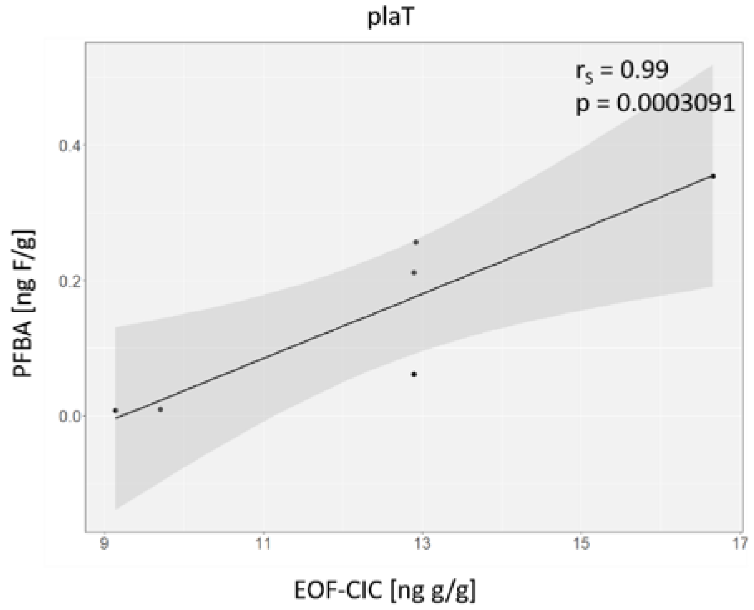

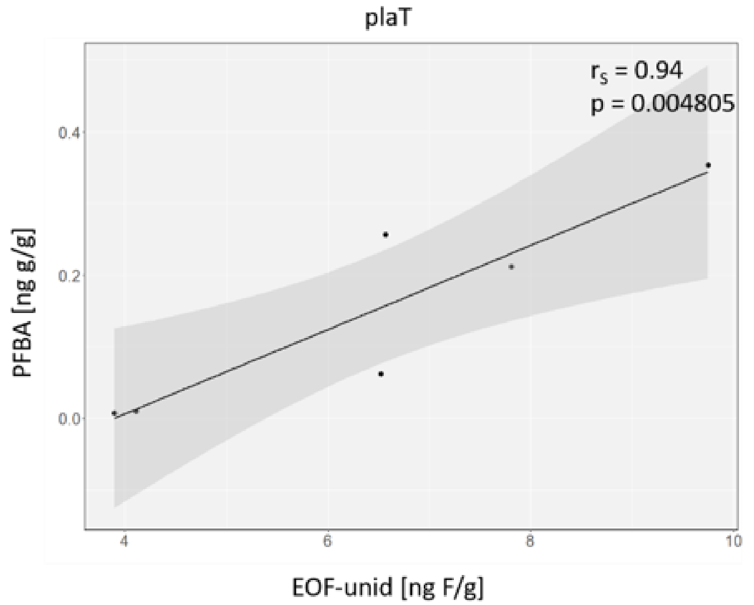

Figure S2: Correlation between PFBA and EOF-CIC, and between PFBA and EOF-unid in plaT.

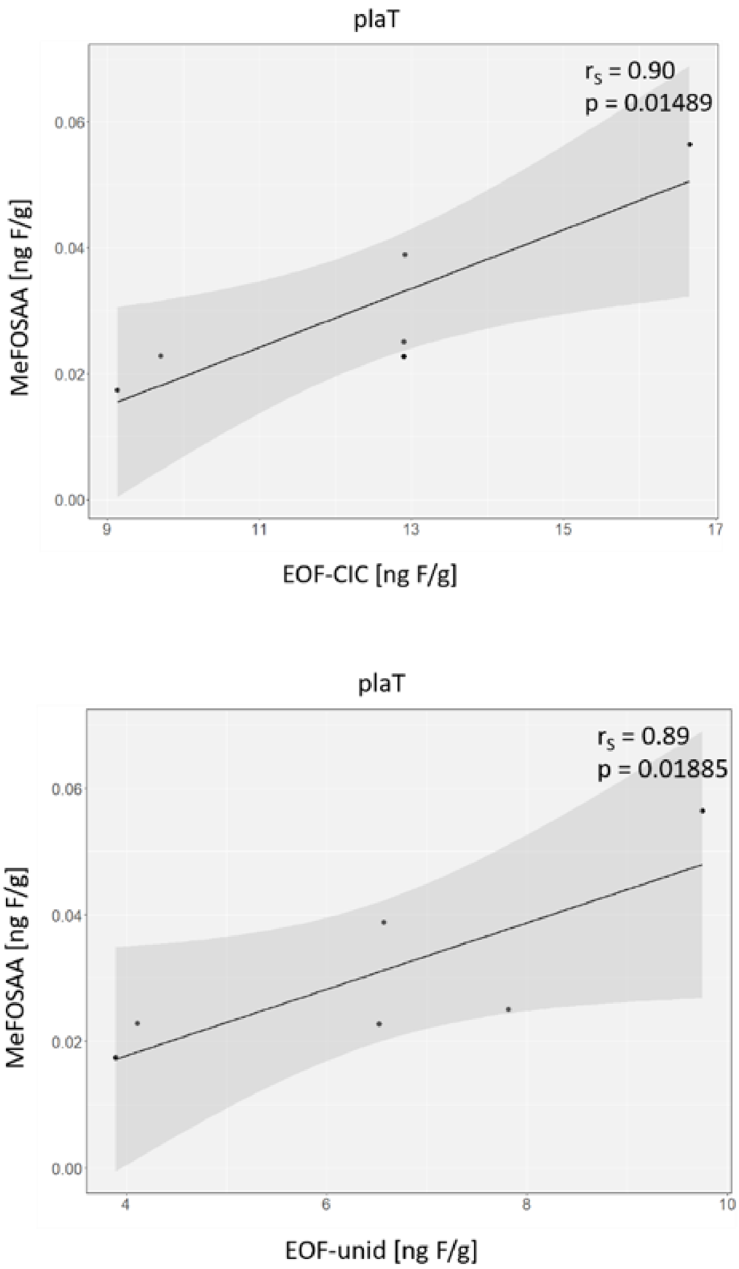

Figure S3: Correlation between MeFOSAA and EOF-CIC, and between MeFOSAA and EOF-unid in plaT.

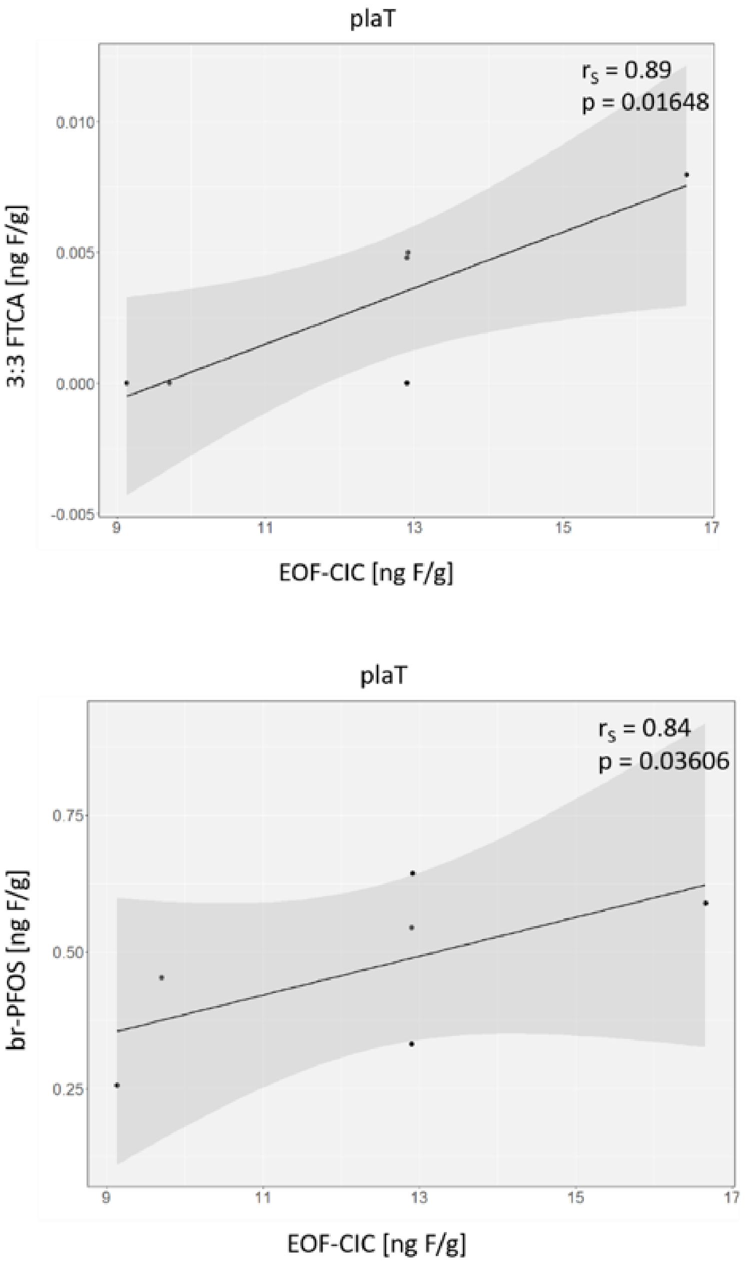

Figure S4: Correlation between 3:3 FTCA and EOF-CIC, and between br-PFOS and EOF-CIC in plaT.

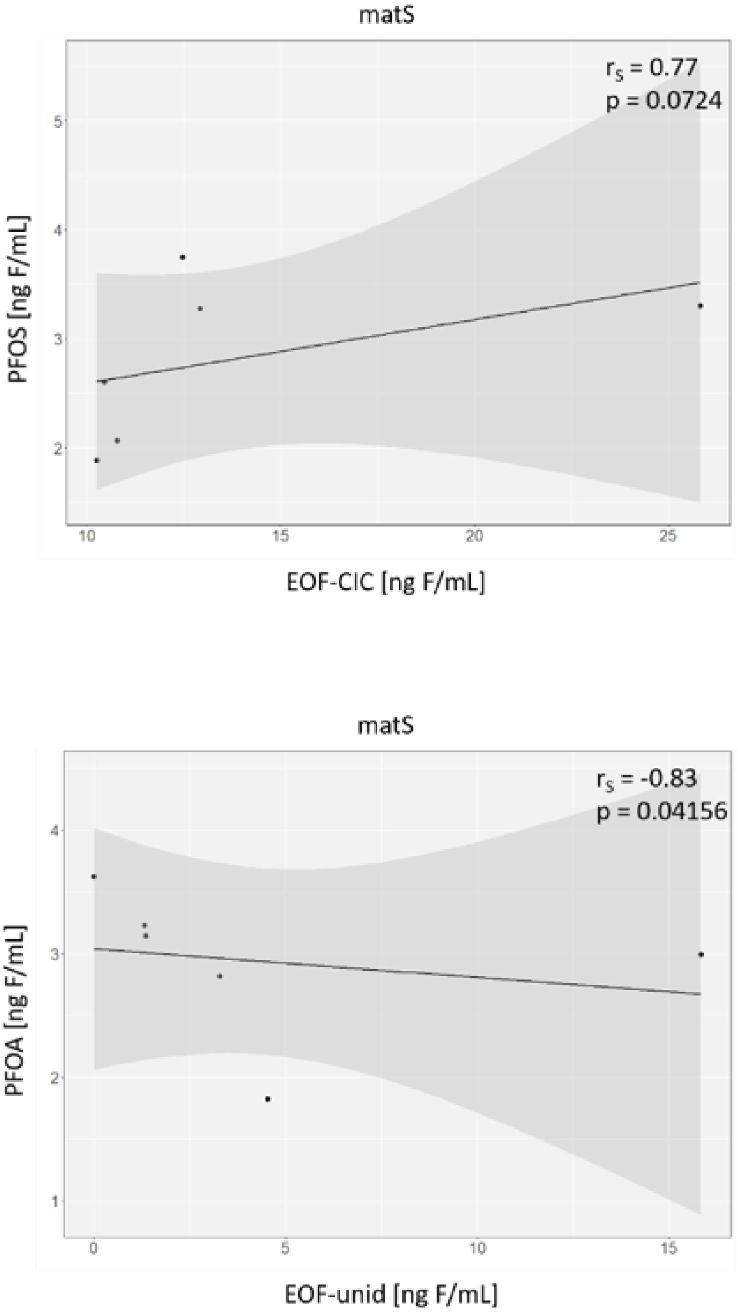

Figure S5: Correlation between PFOS and EOF-CIC, and between PFOA and EOF-unid in matS.

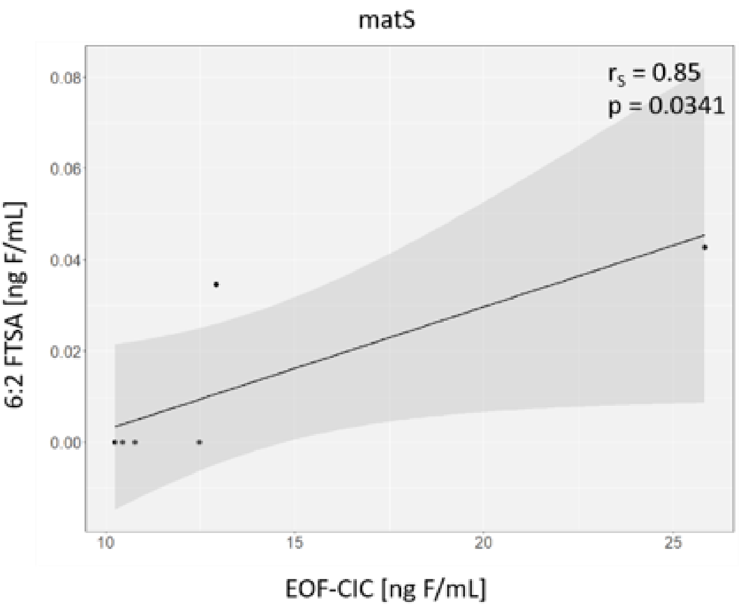

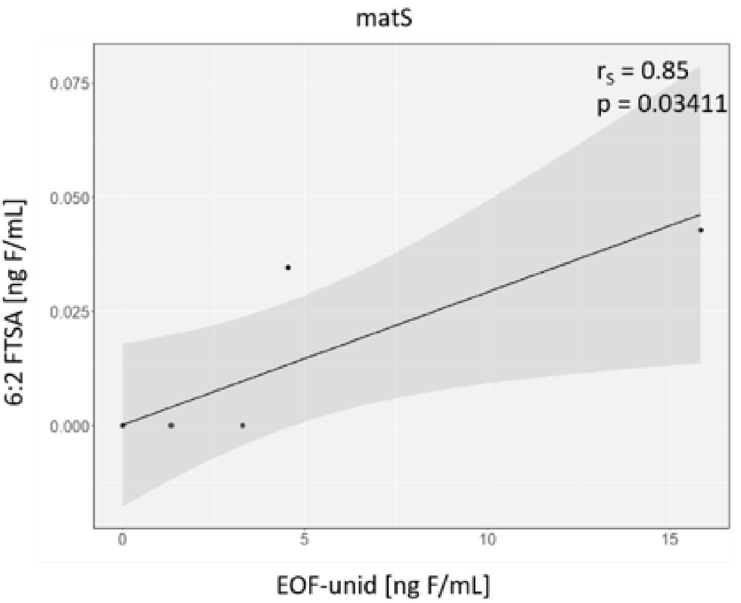

Figure S6: Correlation between 6:2 FTSA and EOF-CIC, and between 6:2 FTSA and EOF-unid in matS.

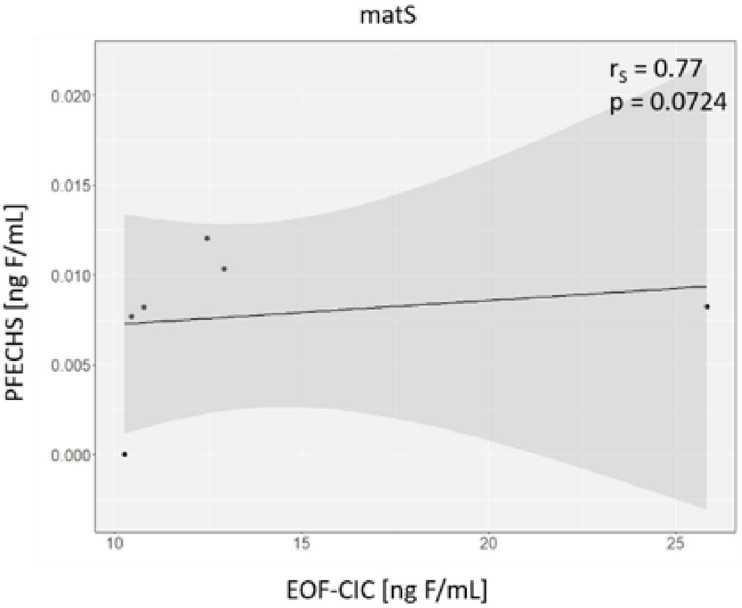

Figure S7: Correlation between PFECHS and EOF-CIC in matS.

Abbreviations

6:2 Cl-PFESA	6:2 chlorinated polyfluorinated ether sulfonate (major F-53B)
6:6 PFPiA	bis(perfluorohexyl)phosphinate (bis(perfluorohexyl)phosphinic acid)
6:8 PFPiA	perfluorohexyl-perfluorooctyl phosphinate (perfluorohexyl-perfluorooctyl phosphinic acid)
8:2 Cl-PFESA	8:2 chlorinated polyfluorinated ether sulfonate (minor F-53B)
8:2 monoPAP	8:2 polyfluoroalkyl phosphate monoester
8:8 PFPiA	bis(perfluorooctyl)phosphinate (bis(perfluorooctyl)phosphinic acid)
ADONA	dodecalfluoro-3H-4,8-dioxanonanoate
CIC	combustion ion chromatography
diPAPs	polyfluoroalkyl phosphate diesters
diSAmPAP	(bis-)2-N-ethylperfluorooctane-n-sulfonamido-ethyl-phosphate
EOF	extractable organofluorine
ESI	electrospray ionization
EtFOSA	N-ethyl-perfluoro-n-octane sulfonamide
EtFOSAA	N-ethyl-perfluoro-n-octane sulfonamido acetic acid
EtFOSE	N-ethyl-perfluoro-n-octane sulfonamido ethanol
FBSA	perfluoro-n-butane sulfonamide
FHxSA	perfluoro-n-hexane sulfonamide
FOSA	perfluoro-n-octane sulfonamide
FOSAs	perfluoroalkyl sulfonamides
FOSEs	perfluoroalkyl sulfonamidoethanols
FTCAs	fluorotelomer carboxylate (fluorotelomer carboxylic acids)

FTOHs	fluorotelomer alcohols
FTSAs	fluorotelomer sulfonate (fluorotelomer sulfonic acids)
FTUCAs	fluorotelomer unsaturated carboxylates (fluorotelomer unsaturated carboxylic acids)
fTW	filtered-tap-water
HF	hydrogen fluoride
HFPO-DA	hexafluoropropylene oxide dimer acid (GenX)
HPLC-MS/MS	high-performance liquid chromatography tandem-mass spectrometry
MeFBSA	N-methyl-perfluorobutane sulfonamide
MeFHxSA	N-methyl-perfluoro-n-hexane sulfonamide
MeFOSA	N-methyl-perfluoro-n-octane sulfonamide
MeFOSAA	N-methyl-perfluoro-n-octane sulfonamido acetic acid
MeFOSE	N-methyl-perfluoro-n-octane sulfonamido ethanol
monoPAPs	polyfluoroalkyl phosphate monoesters
OBS	p-perfluorous noneoxybenzenesulfonate
PAPs	polyfluoroalkyl phosphate esters
PASF	perfluoroalkyl sulfonyl fluoride
PFAAs	perfluoroalkyl acids
PFBA	perfluoro-n-butane carboxylate (perfluoro-n-butanoic acid)
PFBS	perfluoro-n-butane sulfonate (perfluoro-n-butane sulfonic acid)
PFCAs	perfluoroalkyl carboxylates (perfluoroalkyl carboxylic acids)
PFDA	perfluoro-n-decane carboxylate (perfluoro-n-decanoic acid)
PFDoDA	perfluoro-n-dodecane carboxylate (perfluoro-n-dodecanoic acid)
PFDoDS	perfluoro-n-dodecane sulfonate (perfluoro-n-dodecane sulfonic acid)

PFDPA	perfluorodecyl phosphonate (perfluorodecyl phosphonic acid)
PFDS	perfluoro-n-decane sulfonate (perfluoro-n-decane sulfonic acid)
PFECAs	perfluoroalkyl ether carboxylates (perfluoroalkyl ether carboxylic acids)
PFECHS	perfluoro-4-ethylcyclohexane sulfonate
PFESAs	perfluoroalkyl ether sulfonates (perfluoroalkyl ether sulfonic acids)
PFHpA	perfluoro-n-heptane carboxylate (perfluoro-n-heptanoic acid)
PFHpS	perfluoro-n-heptane sulfonate (perfluoro-n-heptane sulfonic acid)
PFHxA	perfluoro-n-hexane carboxylate (perfluoro-n-hexanoic acid)
PFHxDA	perfluoro-n-hexadecane carboxylate (perfluoro-n-hexadecanoic acid)
PFHxPA	perfluorohexyl phosphonate (perfluorohexyl phosphonic acid)
PFHxS	perfluoro-n-hexane sulfonate (perfluoro-n-hexane sulfonic acid)
PFNA	perfluoro-n-nonane carboxylate (perfluoro-n-nonanoic acid)
PFNS	perfluoro-n-nonane sulfonate (perfluoro-n-nonane sulfonic acid)
PFOA	perfluoro-n-octane carboxylate (perfluoro-n-octanoic acid)
PFOcDA	perfluoro-n-octadecane carboxylate (perfluoro-n-octadecanoic acid)
PFOPA	perfluorooctyl phosphonate (perfluorooctyl phosphonic acid)
PFOS	perfluoro-n-octane sulfonate (perfluoro-n-octane sulfonic acid)
PFPAs	perfluoroalkyl phosphonate (perfluoroalkyl phosphonic acids)
PFPeA	perfluoro-n-pentane carboxylate (perfluoro-n-pentanoic acid)
PFPeS	perfluoro-n-pentane sulfonate (perfluoro-n-pentane sulfonic acid)
PFPiAs	perfluoroalkyl phosphinates (perfluoroalkyl phosphinic acids)
PFSAs	perfluoroalkyl sulfonates (perfluoroalkane sulfonic acids)

PFTeDA	perfluoro-n-tetradecane carboxylate (perfluoro-n-tetradecanoic acid)
PFTrDA	perfluoro-n-tridecane carboxylate (perfluoro-n-tridecanoic acid)
PFUnDA	perfluoro-n-undecane carboxylate (perfluoro-n-undecanoic acid)
POPs	persistent organic pollutants
SPE-HLB	solid phase extraction with hydrophilic lipophilic balance
SPE-WAX	solid phase extraction with weak anion exchange
TTEs	transplacental transfer efficiencies
UPLC	ultra-performance liquid chromatography

References

(1) Yeung, L. W. Y.; Mabury, S. A. Are Humans Exposed to Increasing Amounts of Unidentified Organofluorine. **2016**, *Environmental Chemistry*, 102–110.

(2) Miaz, L. T.; Plassmann, M. M.; Gyllenhammar, I.; Bignert, A.; Sandblom, O.; Lignell, S.; Glynn, A.; Benskin, J. P. Temporal Trends of Suspect- and Target-per/Polyfluoroalkyl Substances (PFAS), Extractable Organic Fluorine (EOF) and Total Fluorine (TF) in Pooled Serum from First-Time Mothers in Uppsala, Sweden, 1996–2017. **2020**, *Environmental Science Processes and Impacts 22*, 1071–1083. https://doi.org/10.1039/C9EM00502A.

(3) Yeung, L. W. Y.; Miyake, Y.; Taniyasu, S.; Wang, Y.; Yu, H.; So, M. K.; Jiang, G.; Wu, Y.; Li, J.; Giesy, J. P.; Yamashita, N.; Lam, P. K. S. Perfluorinated Compounds and Total and Extractable Organic Fluorine in Human Blood Samples from China. **2008**, *Environmental Science and Technology 42 (21)*, 8140–8145. https://doi.org/10.1021/es800631n.

(4) Miyake, Y.; Yamashita, N.; Rostkowski, P.; Taniyasu, S.; Lam, P. K. S.; Kannan, K. Trace Analysis of Total Fluorine in Human Blood Using Combustion Ion Chromatography for Fluorine: A Mass Balance Approach for the Determination of Known and Unknown Organofluorine Compounds. **2007**, *Journal of Chromatography A 1154*, 214–221.
